# Methods for a bioengineered 3D human brain-like tissue model of neuroregeneration after traumatic brain injury

**DOI:** 10.4103/NRR.NRR-D-24-00497

**Published:** 2025-05-06

**Authors:** Marly Coe, Sydni Rosenfeld, Celia Byrne, Volha Liaudanskaya, David L. Kaplan

**Affiliations:** 1 Department of Biomedical Engineering, Tufts University, Medford, MA, USA; 2 Department of Biomedical Engineering, University of Cincinnati, Cincinnati, OH, USA

**Keywords:** 3D model, excitotoxicity, glial cells, human brain, *in vitro* model, neurodegeneration, neuronal networks, regeneration, tissue engineering, traumatic brain injury

## Abstract

Traumatic brain injury causes permanent cell death and can lead to long-term cognitive dysfunction, with no available treatments to repair the damaged brain tissue. Methods to track and understand traumatic brain injury in humans are severely limited by the inaccessibility of living brain tissue, creating a need for *in vitro* model systems to study cellular mechanisms of degeneration and regeneration following injury. Here we describe methods to establish a 3D human brain tissue model, consisting of a silk-collagen composite scaffold seeded with human neurons, astrocytes, and microglia, to study neuro-regeneration after traumatic brain injury. Step-by-step fabrication, injury, and analytical assessments of the 3D “triculture” system are described. Using this tissue model system, we demonstrate that glial cells promote regeneration of neuronal networks within the injury site over several weeks post-injury. Further, we found that regenerating networks in the 3D triculture tissues did not secrete early markers of neurodegenerative disease, but displayed signs of excitatory/inhibitory imbalance, suggesting that pro-regenerative treatments for traumatic brain injury in the future may need to direct cell differentiation to promote proper function. The mechanical stability of this model system enables physiologically relevant impact injury and long-term culture capability, while its modular design enables modification of cell contents, extracellular matrix composition, and scaffold properties. This adaptability could allow the integration of patient-derived cells and genetic modifications to bridge research and clinical applications focused on personalized targeted therapies. This *in vitro* system provides a valuable platform for accelerating therapeutic advancements in traumatic brain injury and neurodegenerative disorders, ultimately improving patient outcomes.

## Graphical abstract

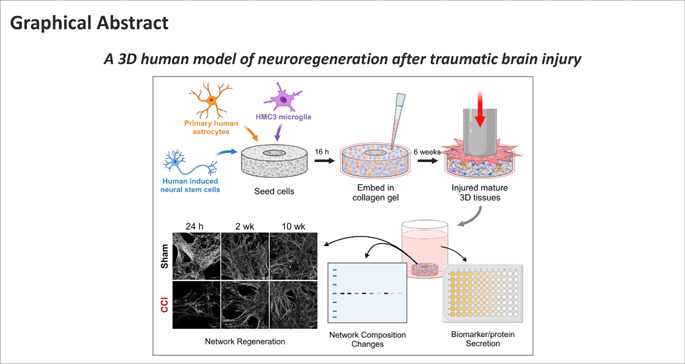

## Introduction

Traumatic brain injury (TBI) affects an estimated 69 million people worldwide each year (Dewan et al., 2018), and is the leading cause of death and disability in young people under 45 (Sun, 2014; Khellaf et al., 2019), with many TBI patients suffering from long-term cognitive impairment or neuropsychiatric disability (Lowenstein, 2009; Kumar et al., 2018). Despite the prevalence of TBI, effective treatments to restore lost neuronal function remain virtually nonexistent, and post-injury care is largely palliative. Pathophysiology of TBI consists of primary tissue damage caused at the time of injury, followed by progressive secondary damage, including cell necrosis and apoptosis, inflammation, excitotoxicity, and metabolic dysregulation (Rauchman et al., 2023) over days, weeks, or even months after injury. The complex molecular mechanisms mediating secondary damage progression in humans are still poorly understood, with even more uncertainty regarding the capacity of damaged neuronal networks to regenerate after injury, as intrinsic mechanisms for self-repair are severely limited in neurons of the central nervous system (Nagappan et al., 2020).

Certain cell populations in the mammalian brain maintain a multipotent state throughout adulthood (Sun, 2014), and increased proliferation, migration, and differentiation of these neural stem cells have been observed in response to injury in both rodent and post-mortem human brain tissues (Zheng et al., 2013; Sun, 2014; Lu et al., 2017; Anderson et al., 2020). However, endogenous neurogenesis is insufficient to replace all neurons lost to injury, and many of these newborn progenitors die or fail to integrate into functional neural networks, preventing complete recovery of damaged brain regions (Zamproni et al., 2021).

To develop new methods to facilitate the repair of damaged brain tissue after TBI, a better understanding of the endogenous cell-cell interactions and molecular mechanisms mediating neuroregeneration is needed. Due to the inaccessibility of living human brain tissue, many TBI studies have relied on animal models such as mice and rats, but the translatability of these studies to human systems may be limited by the inherent differences between rodent and human brains (Burns et al., 2015; Galatro et al., 2017; Hodge et al., 2019). To address this gap, our lab has previously developed a 3D human brain-like tissue model, comprised of a porous silk scaffold seeded with neural cells and embedded in a collagen gel, for the study of neuropathological conditions such as Parkinson’s disease (Fiore et al., 2022), Alzheimer’s and neurodegenerative diseases (Cairns et al., 2020; Rouleau et al., 2020b), and TBI (Liaudanskaya et al., 2020, 2023; Rouleau et al., 2020a; Snapper et al., 2023).

In the present study, we sought to establish this human tissue model as a versatile platform for the long-term *in vitro* study of neuro-regeneration after TBI, with a focus on methods and analytical outcomes to provide a guide for broader use by the community. Toward this goal, we describe methods for 3D tissue scaffold construction, cell seeding, culture, mechanical damage, and assessments of long-term cell responses and regenerative outcomes. After seeding the system with a combination of human neurons, microglia, and astrocytes, we observed glia-driven regeneration of neuronal networks within the injury site at chronic time points post-injury. While regenerated networks did not exhibit a neurodegenerative secretory profile during the study timeframe, there appeared to be a shift towards an excitotoxic phenotype several weeks after injury. The methods to establish and utilize the 3D model presented here provide a tunable *in vitro* platform to investigate pro-regenerative mechanisms in the human brain, and to identify possible points for pharmacological intervention to improve long-term cognitive outcomes after TBI.

## Methods

### Ethics statement

All work in this manuscript was performed *in vitro* using commercially available cell lines. No human or animal subjects requiring ethical approval were involved.

### Cell culture

#### Mouse embryonic fibroblasts

Mouse embryonic fibroblasts (MEFs) were used as a feeder cell layer for human induced neural stem cells (iNSCs) as described previously (Liaudanskaya et al., 2023). Briefly, MEFs (Cat# SCRC-1008, ATCC, Manassas, VA, USA) were seeded into cell culture dishes coated with 0.1% gelatin and cultured in DMEM + GlutaMAX (Cat# 10569010, Gibco, Grand Island, NY, USA) supplemented with 10% heat-inactivated fetal bovine serum (HI-FBS; Cat# A5256801, Gibco) and 1% antibiotic-antimycotic (Cat# 15240062, Gibco). MEFs were expanded into new gelatin-coated plates when they reached approximately 90% confluence. Prior to iNSC seeding, MEFs were allowed to reach 100% confluence and then mitotically inactivated with 10 μg/mL mitomycin-C (Cat# M4287, MilliporeSigma, Milwaukee, WI, USA) for 3 hours at 37°C. After washing three times with Dulbecco’s phosphate-buffered saline (DPBS), inactivated MEFs were maintained in MEF media for up to 7 days, or immediately seeded with iNSCs.

#### Human induced neural stem cells

iNSCs are a human neural progenitor line derived previously from neonatal foreskin fibroblasts through direct reprogramming (Cairns et al., 2016). iNSCs were cultured according to established protocols (Cairns et al., 2016; Liaudanskaya et al., 2023) using Knockout DMEM (Cat# 10829018, Gibco) supplemented with 20% KnockOut SR XenoFree Medium (Cat# 12618013, Gibco), 1% GlutaMAX (Cat# 35050061, Gibco), 1% antibiotic-antimycotic, 0.1 mM β-mercaptoethanol (Cat# 21985023, Gibco), and 20 ng/mL recombinant human fibroblast growth factor-basic (bFGF; Cat# PHG0261, Gibco). Briefly, confluent iNSC colonies were harvested by 2-minute incubation with TrypLE (Cat# 12604021, Gibco) at 37°C, then centrifuged at 1800 × *g* for 2 minutes. For subsequent passaging, cell pellets were gently resuspended in iNSC media to maintain colony morphology and added to a layer of mitotically inactivated MEFs. Media was changed daily to maintain the progenitor state (iNSCs spontaneously differentiate in the absence of bFGF). For seeding into 3D scaffolds, cell pellets were vigorously resuspended in complete Neurobasal media to create a single-cell suspension.

#### Primary human astrocytes

Primary human astrocytes were purchased from ScienCell Research Laboratories (Carlsbad, CA, USA; Cat# 1800) and cultured according to manufacturer-recommended protocols using ScienCell Astrocyte Medium containing 2% FBS, 1% penicillin-streptomycin, and 1% Astrocyte Growth Supplement (Cat# 1801, ScienCell Research Laboratories). Briefly: cell culture dishes were coated with 20 μg/mL poly-L-lysine (PLL; MilliporeSigma) for at least 1 hour at 37°C. Astrocytes were harvested at 90% confluency using 0.25% Trypsin/EDTA, centrifuged at 100 × *g* for 5 minutes, then resuspended in complete astrocyte media and added to PLL-coated dishes. Media was changed every 2–3 days.

#### Production of stable mCherry astrocytes

Pre-made mCherry lentiviral particles containing a puromycin resistance cassette were purchased from GeneCopoeia (Rockville, MD, USA, Cat# LP441-025). A new vial of primary human astrocytes was thawed and expanded as described in the section of “primary human astrocytes,” and seeded at passage 2 into a PLL-coated 6-well plate at 400,000 cells/well. The next day, lentiviral particles were diluted to multiplicity of infection (MOI) = 2 or MOI = 1 in complete astrocyte media containing 5 µg/mL polybrene (Cat# TR1003G, MilliporeSigma). Culture media was replaced with lentiviral (or mock) media and the plate was incubated at 37°C for 24 hours. Transduced wells were each expanded into a PLL-coated T75 culture flask and cultured for another 48 hours before adding puromycin (Cat# J67236XF, ThermoFisher Scientific, Waltham, MA, USA) to the culture media at a concentration of 2 µg/mL. Puromycin media was changed every 2–3 days until cells reached 90% confluence, at which point they were expanded into T175 flasks. After 12 days of antibiotic selection, all mock control astrocytes were dead. mCherry expression in antibiotic-selected astrocytes was confirmed by fluorescence microscopy using a Keyence BZ-X710 (Keyence Corporation, Itasca, IL, USA), and puromycin was removed from the culture media. When T175 flasks reached 90% confluence, all mCherry^+^ astrocytes were trypsinized, combined, and frozen at passage 5.

#### HMC3 microglia

The HMC3 human microglia cell line (Cat# CRL-3304, ATCC) was cultured according to ATCC recommended protocols using EMEM (Cat# 30-2003, ATCC) supplemented with 10% HI-FBS and 1% antibiotic-antimycotic. Cells were harvested at 80%–90% confluency using 0.25% Trypsin/EDTA, centrifuged at 150 × *g* for 5 minutes, then resuspended in complete media and split into new 15 cm culture dishes. Cells were cultured at 37°C and 5% CO_2_ with media changes every 3–4 days.

### Silk scaffold preparation

The preparation of porous silk sponges has previously been described in detail (Rockwood et al., 2011; Dingle et al., 2021). Briefly: whole Bombyx mori silk cocoons (Tajima Shoji Co., Yokohama, Japan) were cut and boiled in 0.02 M Na_2_CO_3_ (MilliporeSigma) solution for 30 mins to remove sericin protein. Degummed silk fibroin fibers were then washed and dried overnight. Dry silk fibroin was dissolved in 9.3 M LiBr (MilliporeSigma) for 4 hours at 60°C, and the fibroin solution was then dialyzed in water for 2 days to remove LiBr. Salt-leached silk sponges were formed by pouring 425–500 μm NaCl crystals (MilliporeSigma) into a 10 cm petri dish containing 6% silk fibroin solution and curing at room temperature for 2 days, followed by 1 hour at 60°C. Cured silk sponges were removed from petri dishes and washed in water for two days to completely remove the salt. Cylinders with 6 mm diameter were cut from 10 cm silk sponges using a biopsy punch, and then cut to 1.5 mm height with a razor blade. A 2 mm diameter central window was cut using a biopsy punch, producing a silk “donut” tissue scaffold. Scaffolds were placed in deionized water and autoclaved (20 minutes, liquid cycle) to sterilize, and then stored at 4°C for up to 3 months.

### 3D brain-like triculture tissue preparation

#### Scaffold coating

Two days prior to cell seeding, sterile silk scaffolds were transferred to a 6-well tissue culture plate (50 scaffolds per well) in a biosafety cabinet. Poly-L-ornithine (7 mL of 10 μg/mL, Cat# A-004-C, MilliporeSigma) in water was added to each well and the plate was incubated overnight at 37°C. The next day each well was aspirated and washed three times with DPBS. Laminin (7 mL of 5 μg/mL, Cat# 11243217001, MilliporeSigma) in DMEM/F12 basal media (Cat# 11039021, Gibco) was added to each well of the plate and scaffolds were coated overnight at 4°C. The next morning the plate was warmed to 37°C before cell seeding.

#### Cell seeding

Complete Neurobasal media was prepared with Neurobasal Medium (Cat# 21103049, Gibco), 1% GlutaMAX, 1% antibiotic-antimycotic, 1X B-27 supplement (Cat# 17-504-044, Gibco), and 1% Astrocyte Growth Supplement (Cat# 1852, ScienCell Research Laboratories). On the day of seeding, iNSCs, astrocytes, and HMC3 microglia were harvested at 100% confluency according to the individual protocols described above. Cells were pelleted, resuspended in complete Neurobasal media, and counted. iNSCs, astrocytes, and microglia were combined at a ratio of 2:0.5:0.1 million, and tubes containing cell mixtures were placed on ice. Coated silk scaffolds were transferred into each well of a 96-well plate using a vacuum manifold to remove excess liquid. Cell mixtures were then pelleted and resuspended in complete Neurobasal media such that 40 μL of cell suspension contained 2 million iNSCs, 500,000 astrocytes, and 100,000 microglia. Using a multi-channel pipettor, 40 μL of cell suspension was pipetted evenly onto each semi-dry scaffold, and the scaffolds were incubated at 37°C for 30 minutes to allow cell attachment. Then an additional 200 μL of complete Neurobasal media was added to each well and plates were returned to the incubator overnight.

#### Collagen embedding

The day after cell seeding, scaffolds were embedded in a collagen hydrogel. Cultrex 3D Culture Matrix Rat Collagen type I (Cat# 344702001, R&D Systems, Minneapolis, MN, USA) was diluted to a final concentration of 3 mg/mL with sterile filtered 10X PBS (Cat# 6506-1L, MilliporeSigma), complete Neurobasal media, and NaOH (Cat# S2770-100ML, MilliporeSigma) to neutralize the acid-solubilized collagen. Calculated volumes of 10X PBS, NaOH, and Neurobasal media were first added to a 15 mL conical tube on ice. Cell-seeded scaffolds were then transferred with sterile tweezers to a new 96-well plate and gently pressed flat onto the bottom of each well. Ice-cold collagen was added to the cold PBS/NaOH/media mixture, and gently mixed to avoid the formation of air bubbles, and the pH was checked using pH indicator strips (Cat# 13-640-517, Fisher Scientific). Once neutralized, the collagen mixture was immediately transferred to a reagent reservoir and 100 μL of collagen mixture was added to each scaffold using a multichannel pipette. Working quickly, tweezers were used to gently release any air bubbles trapped beneath scaffolds, and the plate was incubated at 37°C for 30 minutes to promote gelation. 150 μL of complete Neurobasal media was then added to each well, and the plate was returned to the incubator overnight.

#### Long-term culture

After collagen embedding, scaffolds were gently transferred with tweezers to 48-well plates containing 1 mL of complete Neurobasal media per well. Scaffolds were maintained at 37°C and 5% CO_2_ with twice weekly media changes. For media changes, a vacuum manifold was used to aspirate approximately half of the media in each well, and replaced with 0.5 mL of fresh media. 3D brain-like tissues were cultured for 6 weeks prior to injury.

### 3D brain-like tissue injury

Six weeks after seeding, mature 3D brain-like tissues were injured using a Leica MyNeuroLab Impact One controlled cortical impact (CCI) device (Leica Biosystems, Deer Park, IL, USA) following published protocols (Liaudanskaya et al., 2020, 2023), with a media change 24 hours before injury. Briefly, to mimic CCI injury, 3D tissues were transferred with tweezers to a sterile 10 cm dish and injured using a 3 mm flat tip impactor at 6 m/s impact velocity, 0.6 mm penetration depth, and 100 ms dwell time duration. Sham tissues were handled identically but did not receive an impact. Samples were then returned to their culture wells and maintained at 37°C, 5% CO_2_ with twice weekly media changes until their designated time points for analysis. Each experiment consisted of two or three Sham and four CCI samples per timepoint per analysis, and each analysis was performed on at least three experimental replicates.

### Characterization of cell health and injury progression

#### Viability staining

3D cell growth and viability were monitored at 2 and 5 weeks post-seeding using standard Calcein-AM staining protocols. Briefly, using sterile tweezers, 3D tissues were transferred into a 48-well plate containing 500 μL of 5 μg/mL Calcein-AM (Cat# C3100MP, Invitrogen, Carlsbad, CA, USA) in DPBS. Samples were incubated at 37°C for 30–40 minutes in the dark, then proceeded directly to imaging on a Leica TCS SP8 confocal microscope (Leica Microsystems, Wetzlar, Germany) with 488 nm excitation and 517 nm emission wavelengths.

#### Western blotting

3D brain-like tissues for protein analysis were removed from the culture plate, placed in a 1.5 mL microcentrifuge tube, and frozen at –80°C. On the day of protein isolation, 200 μL of RIPA buffer (Cat# 20-188, MilliporeSigma) supplemented with Pierce Protease and Phosphatase Inhibitor cocktail (Cat# A32959, ThermoFisher Scientific) was added to each tube on ice. 3D tissues were then sonicated using a probe sonicator for 20 pulses (1 second on, 1 second off) at 20% amplitude. Sonicated scaffolds were removed from each tube, and the protein concentration in the solution was quantified by Bradford assay (Cat# 23200, ThermoFisher Scientific) at a 1:10 dilution, according to the manufacturer’s protocol.

For Western blots, 10 μg of protein per well was separated under denaturing conditions on Novex WedgeWell 4-20% Tris-Glycine gels (Cat# XP04205BOX, Invitrogen) and transferred to nitrocellulose membranes (Cat# IB23002, Invitrogen) using an iBlot2 transfer system (Invitrogen). Membranes were blocked for 1 hour at room temperature with 5% non-fat dry milk (NFDM; Cell Signaling Technologies, Danvers, MA, USA) in Tris-buffered saline + Tween-20 (TBST; Cat# IBB181X4X1L, Boston BioProducts, Milford, MA, USA), and subsequently stained with primary antibodies diluted in 5% NFDM + 0.05% sodium azide (VWR, Radnor, PA, USA) overnight at 4°C on a plate rocker. The following day, membranes were washed 4 × 10 minutes on a plate rocker at room temperature, and then incubated with HRP-conjugated secondary antibody diluted in 5% NFDM for 1 hour at room temperature on a plate rocker. All primary and secondary antibodies used for Western blots are listed in **Table 1**. Membranes were washed four more times with TBST, then incubated for 5 minutes with either SuperSignal West Pico Plus chemiluminescent substrate (Cat# A34577, ThermoFisher Scientific), used for Tuj1 and Actin detection, or SuperSignal West Atto Ultimate Sensitivity substrate (Cat# A38555, ThermoFisher Scientific), used for all other proteins. Membranes were then placed between clean plastic sheets and imaged using the BioRad ChemiDoc imaging system (BioRad, Hercules, CA, USA). Western blot images were quantified using Fiji (ImageJ) software, and relative protein expression was normalized to actin or Tuj1 signal. Figures represent fold-change of CCI samples over corresponding Sham controls from each time point.

**Table 1:** Western blot antibodies

Antibody	Vendor	Cat#	Dilution
**Primary antibody**			
Mouse anti-Sox2	Abcam (Waltham, MA, USA)	ab79351	1:1000
Rabbit anti-Pax6	Invitrogen (Carlsbad, CA, USA)	42-6600	1:1000
Rabbit anti-Nestin	Abcam	ab105389	1:1000
Rabbit anti-Sox9	Abcam	ab185230	1:1000
Rabbit anti-NMDAR1	Invitrogen	700685	1:500
Rabbit anti-GAD65+67	Abcam	ab183999	1:1000
Mouse anti-MAP2	Abcam	ab11267	1:1000
Rabbit anti-Tuj1	Abcam	ab18207	1:20000
Mouse anti-Actin, HRP-conjugated	Abcam	ab20272	1:10000
**Secondary antibody**			
HRP-conjugated anti-rabbit IgG	Cell Signaling Technologies (Danvers, MA, USA)	7074P2	1:10000
HRP-conjugated anti-mouse IgG1	Invitrogen	A10551	1:10000

GAD: Glutamic acid decarboxylase; HRP: horseradish peroxidase; MAP2: microtubule-associated protein 2; NMDAR1: N-methyl-D-aspartate receptor 1.

#### Immunofluorescence staining and image analysis

3D brain-like tissues were transferred to a new 48-well plate containing 500 μL of 4% paraformaldehyde (Cat# 15713S, Electron Microscopy Sciences, Hatfield, PA, USA) + 4% sucrose (Cat# S5-500, Fisher Scientific) solution in DPBS. Tissues were fixed for 1.5–2 hours at room temperature, washed 3 × 10 minutes with PBS to remove residual paraformaldehyde, then stored in DPBS at 4°C until the day of staining.

For immunostaining, 3D tissues were blocked and permeabilized in 250 μL of blocking buffer (4% normal goat serum [Cat# 50062Z, ThermoFisher Scientific] + 0.02% Triton X-100 [Cat# X100-100ML, Sigma-Aldrich, Milwaukee, WI, USA] in DPBS) for 1 hour at room temperature, then blocking buffer was replaced with 250 μL of primary antibody diluted in blocking buffer and incubated overnight at 4°C. The next day, samples were washed 4 × 10 minutes with DPBS on a plate shaker at room temperature. Then 250 μL of fluorophore-conjugated secondary antibody diluted in DPBS was added to each well, and tissues were incubated for 1 hour at room temperature in the dark on a plate shaker. The secondary antibody solution was then replaced with 250 μL of 5 μg/mL 4′,6-diamidino-2-phenylindole (DAPI; Cat# D1306, Invitrogen) solution in DPBS and incubated for 5 minutes at room temperature. Tissues were then washed 4 × 10 minutes with DPBS at room temperature, and stained samples were stored in DPBS at 4°C in the dark until imaging. The primary and secondary antibodies used for immunostaining are listed in **Table 2**.

**Table 2:** Immunostaining antibodies

Antibody	Vendor	Cat#	Dilution
**Primary antibody**			
Mouse anti-Tuj1	Abcam	ab78078	1:1000
Rabbit anti-Tuj1	Abcam	ab18207	1:1000
Chicken anti-MAP2	MilliporeSigma (Milwaukee, WI, USA)	AB5543	1:1000
Rabbit anti-Ki67	Abcam	ab15580	1:1000
Rabbit anti-amyloid fibril (mOC87)	Abcam	ab201062	1:1000
**Secondary antibody**			
Goat anti-mouse IgG2a AF488	Invitrogen	A-21131	1:500
Goat anti-rabbit IgG AF488	Invitrogen	A-11008	1:500
Goat anti-chicken IgY AF488	Invitrogen	A-11039	1:500
Goat anti-chicken IgY AF594	Invitrogen	A-11042	1:500
Goat anti-rabbit IgG AF594	Invitrogen	A-11012	1:500

MAP2: Microtubule-associated protein 2.

For Tuj1 network images used for quantification, images were acquired on a Leica TCS SP8 confocal microscope using a Fluotar VISIR 40X/1.10 water objective, 290.62 μm × 290.62 μm, with 100 z-steps of 1.0 μm in depth. All images within each experiment were acquired with the same laser power and PMT gain settings. One representative imaging region was selected from each 3D tissue, and 3–4 different samples were imaged for each experimental time point. Tuj1^+^ neuronal network density was analyzed via a custom MATLAB image analysis script previously published by our group (Liaudanskaya et al., 2020; Snapper et al., 2023), which calculates Tuj1^+^ pixel density in each z-step before compiling a “network” value for all z-steps combined. Figures show 100-step maximum projection images.

#### Enzyme-linked immunosorbent assay

Protein levels in cell culture supernatants were quantified using enzyme-linked immunosorbent assay (ELISA) kits purchased from ThermoFisher Scientific: phospho-Tau [pT181] (Cat# KHO0631, Invitrogen), total Tau (Cat# KHB0041, Invitrogen), amyloid-beta 40 (Cat# KHB3481, Invitrogen), Amyloid beta 42 Ultrasensitive (Cat# KHB3544, Invitrogen). Assays were run according to the manufacturer’s protocol, except samples were diluted in complete neurobasal medium to minimize matrix effects.

#### Glutamate assay

Glutamate levels in cell culture supernatant were quantified using a colorimetric assay kit purchased from Abcam (Waltham, MA, USA, Cat# ab83389). Assays were run according to the manufacturer’s protocol, except samples and standards were all diluted in complete neurobasal medium instead of assay buffer to account for phenol red absorption at 450 nm in the samples.

### Data analysis and statistics

All data are presented as mean ± standard deviation unless otherwise stated. Due to sample-to-sample variability, statistical outliers were removed using the ROUT test with Q = 5%. Comparisons between means were performed via Welch’s unpaired *t*-test or one-way analysis of variance with Dunnett’s T3 *post hoc* test to compare each mean to the corresponding control. All data represent at least three experimental replicates with 3–4 technical replicates per experiment. Data analysis and statistical calculations were performed using GraphPad Prism 10 software (GraphPad Software, Inc., San Diego, CA, USA).

## Results

### Establishing an *in vitro* 3D human brain-like tissue model for the study of neuro-regeneration

**Figure 1:**
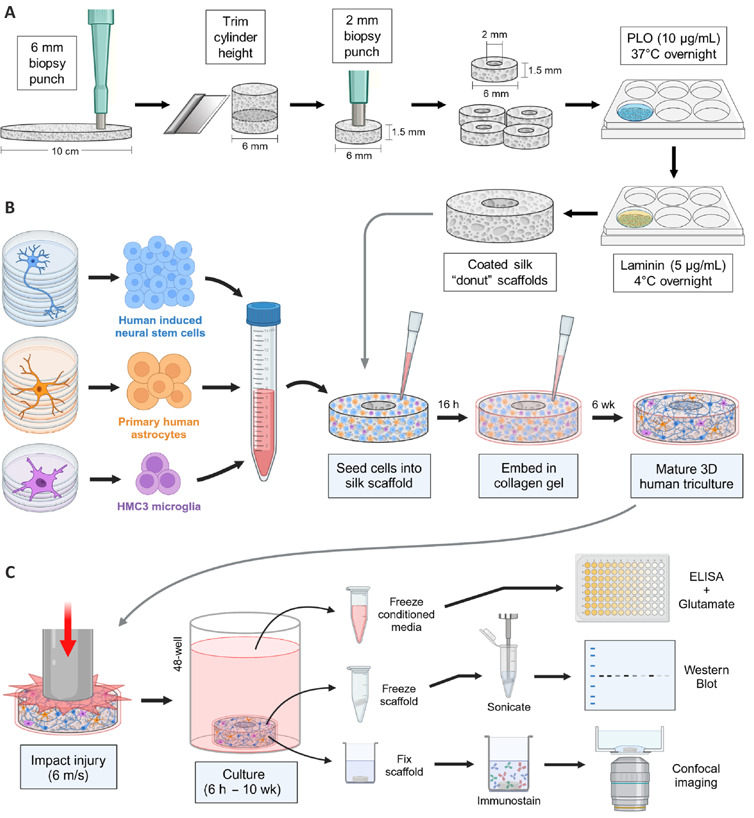
Workflow for the fabrication and analysis of a 3D human triculture model of neuro-regeneration after traumatic brain injury. (A) Silk fibroin sponges were cut into “donut” scaffolds and coated with poly-L-ornithine (PLO) and laminin to promote cell attachment. (B) Human induced neural stem cells (iNSCs), primary human astrocytes, and HMC3 microglia were expanded separately in 2D culture dishes, then dissociated and combined at a 2:0.5:0.1 ratio. Coated silk donut scaffolds were seeded with the cell mixture, embedded in a collagen gel, and cultured for 6 weeks to allow maturation. (C) Mature 3D tissues were injured using a controlled cortical impact device and returned to their culture well for various lengths of time before analysis. Conditioned media was frozen for enzyme-linked immunosorbent assay (ELISA) and glutamate analyses, while whole 3D tissues were frozen for Western blot analysis or fixed for fluorescent immunostaining and imaging. Figure 1B and C was created with BioRender.com.

To study multi-cellular responses following impact injury, 3D brain-like tissues containing a combination of human neurons, astrocytes, and microglia were prepared following recently established protocols (Liaudanskaya et al., 2023; Snapper et al., 2023). Porous silk fibroin “donut” scaffolds were coated with poly-L-ornithine and laminin to promote cell attachment (**Figure 1A**), and seeded with combinations of human iNSCs, human astrocytes, and HMC3 microglia (**Figure 1B**) at a ratio of 2:0.5:0.1 to maintain the approximately 5:1 astrocyte-to-microglia ratio observed in the human cortex (von Bartheld et al., 2016), while accounting for the proliferative capacity of these cells relative to neurons throughout tissue maturation. Seeded scaffolds were then embedded in collagen gel and cultured for six weeks (**Figure 1B**) to allow further cell proliferation, differentiation, and formation of dense neuronal networks (**Additional Figure 1A**) before injury (**Figure 1C**).

**Figure 2:**
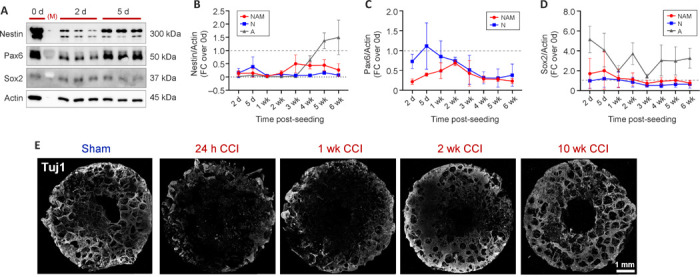
Initial characterization of 3D neuro-regenerative model. (A) Example Western blot images of neural stem cell markers in maturing 3D tissues. (B–D) Quantification of Nestin (B), Pax6 (C), and Sox2 (D) in maturing 3D tissues seeded with induced neural stem cells (blue squares), astrocytes (gray triangles), or the combination of induced neural stem cells, astrocytes and microglia (red circles). Data are presented as mean ± SD, with *n* = 6 for triculture (NAM) and neuron monoculture (N) samples, representing 2 experimental replicates, and *n* = 3 for astrocyte (A) samples from one experiment. (E) Confocal tile-scan images of the neuronal marker Tuj1 in 3D triculture tissues at 24 hours, 1 week, 2 weeks, and 10 weeks post-injury. Scale bar: 1 mm. CCI: Controlled cortical impact.

Western blots for neural stem cell markers Nestin, Pax6, and Sox2 showed that iNSCs quickly began to differentiate after seeding in 3D scaffolds (**Figure 2A–D**). In neuron monocultures (N), Nestin expression dropped to 15.3% ± 15.7% of undifferentiated (0d) expression levels by 2 days post-seeding, and remained low through the 6-week time point when scaffolds were injured **(Figure 2B**). In “triculture” tissues containing neurons, astrocytes, and microglia (NAM), Nestin expression similarly dropped to 13.7% ± 9.2% of 0d expression levels by 2 days post-seeding but then increased again at 3 weeks, corresponding to increasing Nestin expression in astrocyte monoculture tissues (A) around this time (**Figure 2B**). Pax6 expression levels in both N and NAM tissues decreased at later time points post-seeding (**Figure 2C**). Interestingly, Sox2 expression in NAM tissues appeared to increase after seeding into 3D scaffolds, likely due to astrocytes which showed approximately 2- to 4-fold more Sox2 at all time points compared with 0d controls (**Figure 2D**). N tissues, however, generally showed a slight decreasing trend in Sox2 expression out to 6 weeks post-seeding (**Figure 2D**). Altogether these data suggest that iNSCs seeded into 3D brain-like tissues undergo differentiation and maturation before injury, but that a population of these cells does maintain immature, stem-like phenotypes.

At 6 weeks post-seeding, 3D brain-like tissues were injured using a CCI device (**Figure 1C** and **Additional Figure 1B**) with parameters used to mimic moderate TBI in *in vivo* mouse models (Liaudanskaya et al., 2020). As observed in previous studies (Liaudanskaya et al., 2023), neuronal networks within the impact and peri-impact areas were largely destroyed within 24 hours (**Figure 2E**). Surprisingly, however, we found that over several weeks in culture the networks began to spontaneously regenerate, with regeneration progressing from the healthy peripheral regions into the central impact area, and the 3D tissues appeared to be completely recovered by 10 weeks post-injury (**Figure 2E**). Following this observation, we sought to further characterize the regenerative capacity of these 3D brain-like tissues.

### Glia promote regeneration of neuronal networks in injured 3D brain-like tissues

**Figure 3:**
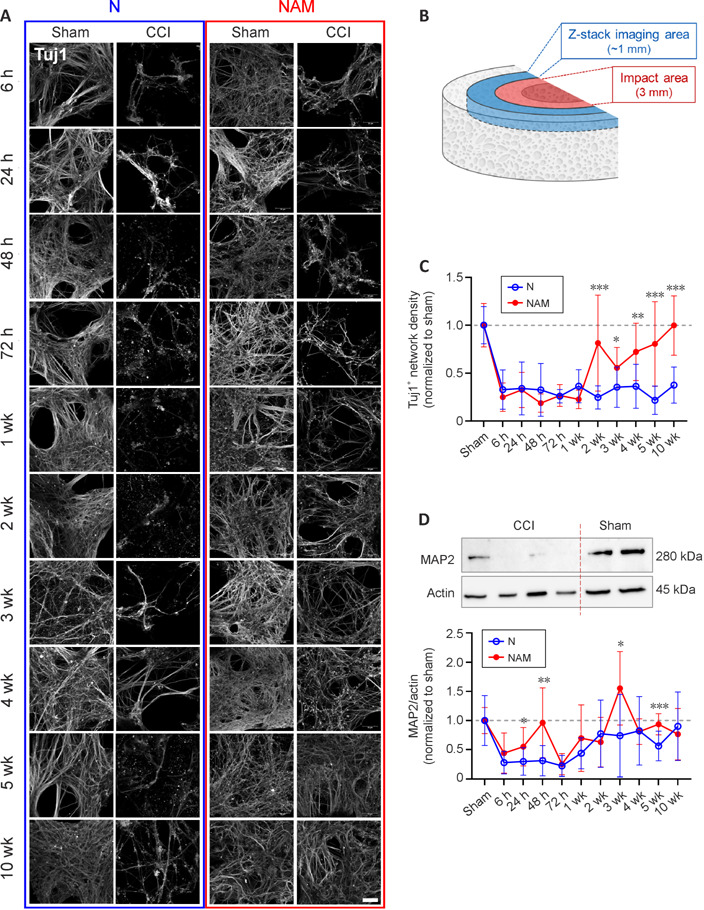
Glial cells increase the regenerative capacity of 3D triculture tissues. (A) Representative confocal images of Tuj1-stained neuron monoculture (N) and triculture (NAM) tissues from 6 hours to 10 weeks post-injury. Scale bar: 50 µm. (B) Schematic diagram of the impact and imaging areas. (C) Quantification of Tuj1^+^ network density from confocal images of N and NAM tissues after injury, *n* = 8–12 N tissues and *n* = 12–16 NAM tissues per timepoint, representing data from 3–4 independent experiments. (D) Example of controlled cortical impact (CCI) *vs.* Sham blot images, and quantification of Western blots for microtubule-associated protein 2 (MAP2) protein in N and NAM tissues after injury, *n* = 12–16 tissues per timepoint from 3–4 independent experiments. All data are presented as mean ± SD. *P < 0.05, **P < 0.01, ***P < 0.001 (Welch’s unpaired *t*-test) between N group and NAM group.

A previous study using 3D brain-like tissues seeded with only iNSCs had shown no signs of neuronal network regeneration by 2 weeks post-injury (Liaudanskaya et al., 2023), while our triculture tissues appeared to begin regenerating at this time (**Figure 2E**). To compare the regenerative capacity of neuronal networks with and without added glial cells, both N and NAM tissues were injured and the resulting neuronal network density was compared at acute, subacute, and chronic timepoints post-injury. Immunostaining for the neuronal marker β-tubulin III (Tuj1) showed that neuronal networks in the injury penumbra (**Figure 3B**) of N tissues had minimal recovery even at 10 weeks post-injury, while NAM tissues displayed robust network regrowth (**Figure 3A**).

MATLAB quantification of Tuj1^+^ pixel density within the confocal z-stack images (Liaudanskaya et al., 2020) revealed that N tissues dropped to 32.9% ± 19.2% of sham network density by 6 hours post-injury and did not increase, remaining at 37.6% ± 18.1% of sham at 10 weeks (**Figure 3C**). The slight increase in network observed in injured N tissues was counteracted by a slight increase in sham network density throughout the 10 week period of observation (**Additional Figure 2**). NAM tissues had a similar network decrease immediately after injury, dropping to 24.9% ± 14.2% of sham by 6 hours, but showed a significant increase over N tissues beginning at 2 weeks post-injury and continuing through 10 weeks, at which point NAM tissues appeared equivalent to shams (**Figure 3C**). Interestingly, NAM tissues did not seem to have the same increase in sham network density seen in the N tissues (**Additional Figure 2**), as later time points (particularly 10 weeks) often had thicker neuronal processes than early time points.

To assess network density in areas of the 3D tissue not visible during imaging, Western blots for the mature dendrite marker microtubule-associated protein 2 (MAP2) were performed on protein lysate from the whole 3D tissue (i.e., no distinction between injury site and peripheral areas), and showed similar trends in N and NAM tissues after injury (**Figure 3D**). MAP2 expression decreased quickly in both N and NAM tissues acutely after injury. However, there appeared to be less dendrite loss in NAM tissues than in N tissues at 24 and 48 hours, perhaps due to supportive functions from the glial cells. MAP2 levels in all tissues began increasing again around 1–2 weeks post-injury, with near complete recovery at 3–4 weeks. NAM tissues may have had slightly higher MAP2 levels at 3 and 5 weeks post-injury (*P* = 0.0174 and *P* = 0.000023, respectively) but appeared equivalent to N tissues again at 10 weeks. These data suggest that, outside of the injury site, bulk regions of the 3D tissue regenerate (or continue growing if the impact did not kill the cells) with similar kinetics in N and NAM tissues. It is important to note here that the iNSCs used in this model can produce both neurons and astrocytes (Cairns et al., 2016), and thus are not truly neuron-only constructs, although N tissues have fewer astrocytes than NAM tissues at the time of injury as evidenced by Western blot for Sox9 (**Additional Figure 3**).

**Figure 4:**
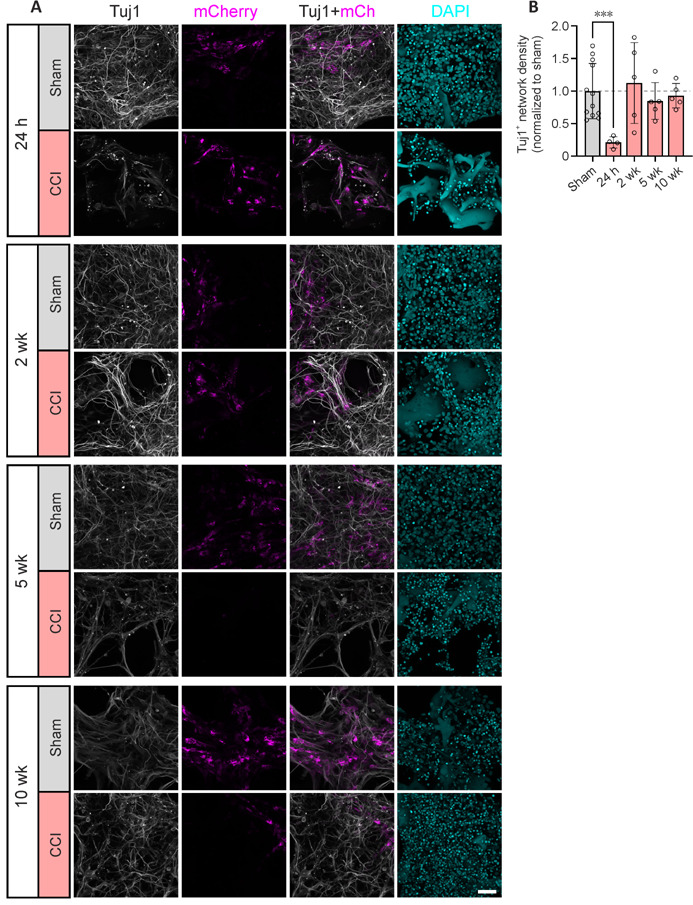
iNSCs are the primary source of regenerating neuronal networks. (A) Representative images of Tuj1^+^ neuronal networks, mCherry^+^ astrocytes, and 4′,6-diamidino-2-phenylindole (DAPI) nuclear staining in triculture tissues with mCherry^+^ astrocytes (N-mChA-M) at 24 hours, 2 weeks, 5 weeks, and 10 weeks after injury. Scale bar: 50 µm. (B) Quantification of Tuj1^+^ network density from confocal images of N-mChA-M tissues after injury. Data are presented as mean ± SD, with *n* = 5 CCI and *n* = 3 Sham for each time point, samples from one experiment. ***P < 0.001 (one-way analysis of variance with Dunnett’s T3 *post hoc* test). CCI: Controlled cortical impact; iNSCs: induced neural stem cells.

Because the primary astrocytes incorporated in the triculture system may have some neurogenic potential, stable mCherry-expressing astrocytes (mChA) were produced to better determine the source of regenerating neurites in the NAM tissues. After impact injury, N-mChA-M triculture tissues regenerated similarly to NAM tissues (**Figure 4**), and mCherry imaging showed that Tuj1^+^ networks were predominantly iNSC-derived. N-mChA-M tissues also revealed that many astrocytes also expressed Tuj1 (consistent with a fetal phenotype (Dráberová et al., 2008)), though in dimmer, wider processes that were visually distinct from most neurons.

### Secreted markers do not indicate a neurodegenerative phenotype in regenerated neuronal networks

**Figure 5:**
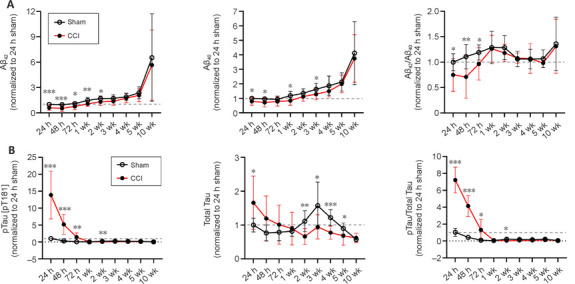
Regenerating 3D tricultures do not show a neurodegenerative secretory profile. (A) ELISA quantification of Aβ_42_, Aβ_40_, and Aβ_42_/Aβ_40_ ratio in cell culture supernatant from triculture (NAM) tissues at 24 hours to 10 weeks post-injury. *n* = 12 Sham and *n* = 16 CCI samples for each timepoint, from 4 independent experiments. (B) ELISA quantification of phosphorylated tau (pTau), total Tau, and pTau/Tau ratio in cell culture supernatant from NAM tissues at 24 hours to 10 weeks post-injury. *n* = 9 Sham and *n* = 12 CCI samples for each time point, from three independent experiments. All data are presented as mean ± SD. *P < 0.05, **P < 0.01, ***P < 0.001 (Welch’s unpaired *t*-test) between Sham group and CCI group. Aβ: Amyloid-beta; CCI: controlled cortical impact; ELISA: enzyme-linked immunosorbent assay.

Patients who experience a TBI have a 2–4 times higher risk of developing dementia later in life (Shively et al., 2012). To assess the health of regenerating neuronal networks, we examined several markers of neurodegenerative disease in 3D triculture tissues after injury. Amyloid-beta 1–40 (Aβ_40_) is the predominant form of beta amyloid protein in the brain, while Amyloid-beta 1–42 (Aβ_42_) is an isoform that is often sequestered in the characteristic amyloid plaques of Alzheimer’s disease patient brains (Graff-Radford et al., 2007), and a lower Aβ_42_/Aβ_40_ ratio in cerebrospinal fluid or plasma has been correlated with cognitive decline (Graff-Radford et al., 2007; Hansson et al., 2007). Using cell culture supernatant as a cerebrospinal fluid analog, ELISAs for secreted Aβ_42_ and Aβ_40_ proteins showed no significant differences between sham and CCI conditions in regenerating tissues out to 10 weeks post-injury (**Figure 5A**). There was, however, slightly lower Aβ_42_ and Aβ_40_ secretion at acute and subacute time points after injury, which may have been due to the decreased neuron density acutely after injury. Interestingly, both sham and CCI conditions showed a steady increase in secreted Aβ levels over time in culture. No plaque-like formations were visible in 3D tissues (**Additional Figure 4**).

Additionally, ELISAs were used to quantify secreted total Tau (tTau) and phosphorylated Tau 181 (pTau181), as these proteins play a central role in the formation of neurofibrillary tangles and have been investigated both as TBI prognostic markers and as biomarkers for neurodegenerative conditions such as Alzheimer’s disease (Riemenschneider et al., 2003; Hampel et al., 2004; Öst et al., 2006; Flavin et al., 2023). pTau181 levels in cell culture supernatant peaked acutely after injury, increasing to 13.9 ± 6.7 times sham levels at 24 hours post-injury, but rapidly decreased to sham levels by 1 week post-injury (**Figure 5B**), around the time that neuronal networks began to regenerate. Total Tau levels followed a similar trend, reaching a 1.66 ± 0.75-fold increase over sham levels at 24 hours post-injury and then returning to sham levels around 72 hours post-injury (**Figure 5B**). Interestingly, tTau secretion in sham, but not CCI, tissues appeared to increase between 1 and 3 weeks post-injury and then decrease again between 3 and 10 weeks, though tTau levels in sham and CCI conditions were equivalent at 10 weeks post-injury. An acute increase in both pTau and tTau levels is a hallmark of TBI that has been observed *in vivo* as well, with concentrations in cerebrospinal fluid generally returning to normal at chronic time points post-injury (Bagnato et al., 2018). An increased pTau/tTau ratio in plasma has been correlated with chronic TBI symptoms (Rubenstein et al., 2017), but no difference was observed between sham and CCI conditions beyond 2 weeks post-injury in 3D triculture tissues.

### Regenerated neuronal networks show excitatory changes in cellular composition

**Figure 6:**
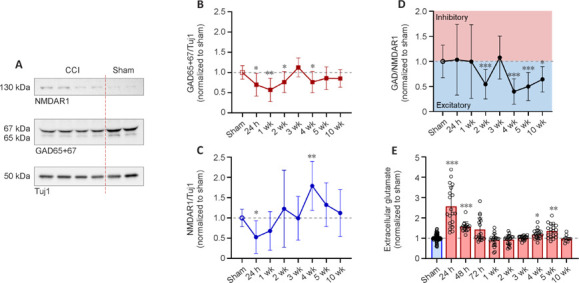
Regenerating neuronal networks show excitatory imbalance. (A) Example of controlled cortical impact (CCI) *vs.* Sham western blot images of excitatory neuron marker NMDAR1, inhibitory neuron marker GAD65+67, and Tuj1 protein as a neuron loading control. (B) Quantification of Western blots for GAD65+67 protein in triculture (NAM) tissues between 24 hours and 10 weeks post-injury. *n* = 12–16 tissues per timepoint, from 3–4 independent experiments. (C) Quantification of Western blots for NMDAR1 protein in NAM tissues between 24 hours and 10 weeks post-injury. *n* = 12–16 tissues per timepoint, from 3–4 independent experiments. (D) Comparison of GAD/NMDAR1 protein ratio (from Western blots in B and C in NAM tissues at various times after injury, as compared with Sham control. (E) Quantification of extracellular glutamate in conditioned cell culture media from NAM tissues between 24 hours and 10 weeks post-injury. *n* = 11 tissues for 10 weeks timepoint, and *n* = 19 tissues for all other timepoints, from 3–4 independent experiments. All data are presented as mean ± SD. *P < 0.05, **P < 0.01, ***P < 0.001 (one-way analysis of variance with Dunnett’s T3 *post hoc* test) between CCI and Sham. ELISA: Enzyme-linked immunosorbent assay.

While secreted proteins did not indicate a clear neurodegenerative phenotype in regenerating neuronal networks, we sought to determine whether the composition of regenerated networks was the same or different from uninjured sham networks. Western blots (**Figure 6A**) revealed that glutamic acid decarboxylase (GAD65+67), expressed by GABAergic inhibitory neurons, decreased to 69.6% ± 26.8% of sham levels 24 hours post-injury, and then further decreased to 57.0% ± 27.7% of sham at 1 week post-injury (**Figure 6B**). While there was some recovery at later time points, GAD65+67 generally remained at or below sham levels out to 10 weeks post-injury. N-methyl-D-aspartate receptor 1 (NMDAR1), expressed by glutamatergic excitatory neurons, showed a similar drop in the acute phase after injury, decreasing to 52.7% ± 38.4% of sham levels at 24 hours (**Figure 6C**). However, NMDAR1 levels followed the opposite trend to GAD65+67, beginning to increase at 1 week and remaining at or above sham levels out to 10 weeks post-injury (**Figure 6C**). Comparison of the GAD/NDMAR1 ratio at each time point showed a trend towards more excitatory neurons in regenerating tricultures, as compared with sham controls (**Figure 6D**).

This trend was further supported by the quantification of glutamate released into the cell culture media (**Figure 6E**). We observed that extracellular glutamate levels spiked acutely after injury, increasing to 2.57 ± 0.95 times sham levels at 24 hours and then rapidly decreasing to 1.57 ± 0.23 times sham at 48 hours and 1.42 ± 0.56 times sham at 72 hours post-injury (**Figure 6E**). Similar trends have been observed via microdialysis in human patients after severe TBI (Chamoun et al., 2010). Glutamate returned to sham levels by 1 week post-injury but began to slowly increase again at 3–4 weeks post-injury, suggesting an increase in excitatory neuron activity or possibly impaired glutamate re-uptake in the regenerating tricultures out to 5 weeks post-injury (**Figure 6E**).

## Discussion

Neuronal networks in the brain are complex 3D structures, and injury can lead to permanent cognitive impairment due to the limited regenerative capacity of these networks. Because living human brain tissue is generally inaccessible, model systems are crucial to understanding how cells respond to and recover from injury. Researchers have often turned to *in vivo* rodent models that recapitulate the complex 3D microenvironment and multi-cellular aspects of brain injury, but this complexity can make it difficult to track specific cellular functions, in addition to the high cost and ethical concerns inherent to *in vivo* models. Moreover, no TBI treatments found to be effective in rodents have yet been successfully translated to the clinic, highlighting the importance of human model systems in bridging the gap between research and clinical practice. Advances in bioengineering have enabled the fabrication of 3D *in vitro* model systems that better mimic *in vivo* cell function in the lab, but very few 3D human models of TBI have so far been published. Of these, even fewer contain both neurons and glial cells, and their application has primarily been limited to acute (hours to days) changes following TBI (Dwyer and Morrison, 2022; Hanna and Pfister, 2022).

Here we have described methods to fabricate and utilize a 3D human brain-like tissue model as a novel *in vitro* platform for the long-term study of neuro-regeneration after TBI. Porous silk “donut” scaffolds were seeded with human neurons, astrocytes, and microglia, and then embedded in a soft collagen gel to allow neurite extension. The donut-shaped scaffold creates distinct “grey matter”- and “white matter”-like regions (donut and central window, respectively) while maintaining the size and shape of the 3D tissue construct over many months in culture, unlike hydrogel-only 3D models that tend to contract over time (Tang-Schomer et al., 2014). Moreover, the 3D tissues are large and mechanically robust enough to withstand CCI injury parameters used in *in vivo* rodent models, an advantage over hydrogel-only models including organoids that require custom injury devices and optimized parameters to mimic TBI (Bar-Kochba et al., 2016; Ramirez et al., 2021; Shi et al., 2021; Loussert-Fonta et al., 2023).

We demonstrated that upon impact injury, the 3D tricultures undergo rapid neuronal network degradation and release glutamate and tau proteins during the acute injury phase – classic hallmarks of TBI *in vivo* (Guerriero et al., 2015; Flavin et al., 2023). Surprisingly, we then found that glial cells supported the spontaneous and robust regeneration of neuronal networks within the injury site over the course of several weeks following injury, highlighting the importance of including glial cells in *in vitro* models of neural injury or disease. Although the source of regeneration in these 3D triculture tissues appears to be a stem-like, mitotically active iNSC population (**Additional Figure 5C**), these findings may have particular relevance to TBI therapies targeting endogenous neural stem cell populations or using exogenous stem cell transplantation. Stem cell transplantation has shown some promise in clinical trials (Adugna et al., 2022), but these treatments often suffer from poor survival and integration of transplanted stem cells within the injured brain lesion (Mahumane et al., 2018). This 3D *in vitro* model may help identify key pro-regenerative glial cell functions that could be induced within the injury site to enhance stem cell survival after transplantation.

Within the 10-week study timeframe we did not observe differences in the secretion of neurodegenerative disease hallmarks from the regenerating networks compared with sham controls. However, TBI patients who develop dementia often do not experience symptoms until later in life, leaving the possibility that longer-term studies are necessary to observe these phenotypes. Importantly, the silk-collagen composite scaffolds remain stable for long periods of time in culture (**Additional Figure 6**) and have been shown to support cell survival for at least 2 years (Rouleau et al., 2020b), so future studies could examine neuronal network phenotypes at 1–2 years post-injury.

Depending on injury severity, approximately 2%–15% of TBI patients develop pharmaco-resistant epilepsy (Ding et al., 2016), and *in vivo* mouse studies have shown that this may be due to GABAergic dysregulation after TBI (Cantu et al., 2015; Witkin et al., 2024). Using Western blots, we found evidence that regenerating networks have a more excitatory neuronal composition compared with sham controls, though further studies are needed to confirm this imbalance on a functional rather than protein level. Future work could utilize electrophysiology (Rouleau et al., 2020a) or adapt previously published methods using the fluorescent GCaMP6f calcium reporter (Dingle et al., 2021) to monitor region-specific changes in the spontaneous electrical activity of neuronal networks over time post-injury. Nonetheless, the preliminary data from this *in vitro* model suggest that the hostile post-TBI microenvironment may not facilitate the regeneration of healthy, balanced networks, which could pose a challenge for future neuro-regenerative methods such as stem cell transplantation studies. If necessary, future *in vitro* studies could focus on methods to specify neural progenitor fate using exogenous growth factors or other small molecules.

As with any model system, the 3D triculture model described here is not without limitations. Neuronal networks in the adult brain are post-mitotic, with reduced plasticity in favor of functional stability (Nagappan et al., 2020), but *in vitro* brain models rely on relatively immature cells to enable their survival during culture and manipulation. The astrocytes used here appear to be of fetal origin (evidenced by Tuj1^+^ staining and previous transcriptomic data (Yin et al., 2019)), and Western blots indicated the presence of an immature iNSC population at the time of injury. While most *in vitro* brain model systems using induced pluripotent stem cell (iPSC)-derived cells, especially organoids, suffer from similar limitations in cell maturity (Mobini et al., 2019; Grenier et al., 2020; Hanna and Pfister, 2022), this fetal phenotype confers higher plasticity and thus limits their translation to adult pathologies including injury, neurodegeneration, and regeneration. Some progress has been made in accelerating the differentiation and aging process of neural stem cells *in vitro*, including the use of specific extracellular matrix (ECM) cues (Rouleau et al., 2023), NOTCH inhibitors or telomerase downregulators (Qian et al., 2019), but development of these methods is still ongoing. For future studies using this 3D triculture model system, it is possible that tissue maturity could be enhanced by extending the culture time before injury by several months, as many iPSC-based models continue to mature over 9–12 months in culture (Cantley et al., 2018; Loussert-Fonta et al., 2023; Rouleau et al., 2023).

Additionally, in this model we used a collagen I hydrogel to create the ECM. While collagen I is cost-effective, has well-characterized and tunable physical properties, and gels under physiological conditions, it is not an abundant component of brain ECM, and a primarily single-protein matrix represents an over-simplified model system. Brain ECM properties can play a critical role in tissue health and disease (Rauti et al., 2020), and altering matrix complexity or composition can affect the differentiation, maturation, and survival of neural cells in 3D (Sood et al., 2019). Thus, future work could incorporate additional brain-specific ECM components such as hyaluronan or even decellularized brain ECM to study their effects on 3D triculture maturation and post-injury regeneration.

Finally, although this 3D triculture system contains microglia, which do not differentiate from neural progenitor cells and are therefore absent from many *in vitro* human brain models, it cannot recapitulate all aspects of TBI-induced neuroinflammation seen *in vivo*. In addition to some functional differences between the immortalized HMC3 microglia cell line and primary human microglia (Dello Russo et al., 2018), the 3D tricultures most notably lack components of the blood-brain barrier, which plays an important role in neuroinflammation and secondary injury progression after TBI (Hubbard et al., 2021). This may cause injury-induced inflammation to resolve more quickly in the model system than *in vivo*, altering pro-regenerative glial cell functions. Thus the inflammatory response of our 3D *in vitro* model could be improved by the use of iPSC-derived microglia (Liaudanskaya et al., 2023), incorporation of a blood–brain barrier component including brain endothelial cells and pericytes, or possibly by post-injury treatment with peripheral blood components including serum, platelets, or leukocytes to exacerbate the neuroinflammatory response.

Despite these limitations, the modular nature of this top-down bioengineered system allows modification of each component to address specific biological questions. The cell seeding process allows control of cell types, sources, and ratios, promoting various combinations of primary cells, cell lines, iPSC-derived cells, fluorescently labeled cells, or even genetically modified cells to investigate specific cellular mechanisms during regeneration. The physical and chemical properties of the scaffold can be tuned by altering the silk porosity or the hydrogel composition. The size of the 3D tissues enables easy handling for injury and analysis, while the structural integrity of the silk scaffold allows physiologically relevant impact injury and long-term culture of 2 years or more – a necessity for long-term neuroregeneration studies.

Overall, this 3D tissue model provides a unique human platform for screening neuroprotective or pro-regenerative drugs and biologics including extracellular vesicles, or for improving cell therapies such as stem cell transplantation or endogenous cell transdifferentiation that are difficult to characterize in complex *in vivo* environments. While *in vivo* models are important to our understanding of biological systems, 3D human *in vitro* models will play a critical role in facilitating the translation of pre-clinical studies to effective clinical treatments. Neuro-regenerative mechanisms explored via the 3D brain-like tissue model presented here may inform future efforts to develop reparative treatments not just for TBI, but for other neurodegenerative conditions including Alzheimer’s disease, Huntington’s disease, or stroke.

## Additional files:

***Additional Figure 1:*** *Development and injury of 3D brain-like tissues.*

Additional Figure 1 Development and injury of 3D brain-like tissues.
(A) Calcein-AM live cell staining of triculture (NAM) and neuron monoculture (N) tissues at 2 weeks and 5 weeks post-seeding to confirm proliferation and differentiation of cells in 3D prior to injury. Scale bars: 100 μm. (B) Controlled cortical impact device setup used for injury of 3D brain-like tissues.

***Additional Figure 2:*** *Un-normalized Tuj1*^*+*^ *network values show changes in Sham networks over time.*

Additional Figure 2 Un-normalized Tuj1^+^ network values show changes in Sham networks over time.
Raw Sham and controlled cortical impact (CCI) “network” values quantified from confocal images of Tuj1-stained (A) neuron monoculture (N) tissues (*n* = 4-6 for Sham and *n* = 8-12 for CCI, from 2-3 independent experiments) and (B) triculture (NAM) tissues (*n* = 6-8 for Sham and *n* = 12-16 for CCI, from 3-4 independent experiments) after injury. Data presented as mean ± SD. *P < 0.05, **P < 0.01, ***P < 0.001 (Welch’s unpaired *t*-test) between Sham group and CCI group.

***Additional Figure 3:*** *Comparison of astrocyte content in NAM and N tissues.*

Additional Figure 3 Comparison of astrocyte content in NAM and N tissues.
(A) Western blot images of astrocyte marker Sox9 and Actin loading control in 6-week-old triculture (NAM) and neuron monoculture (N) tissues. Each lane represents protein from an individual NAM or N sample. (B) Quantification of Sox9/Actin Western blot signal from A. Data presented as mean ± SD. *P < 0.05 (two-tailed *t*-test) between NAM and N.

***Additional Figure 4:*** *Regenerated networks do not show amyloid plaque formation.*

Additional Figure 4 Regenerated networks do not show amyloid plaque formation.
Representative images of triculture (NAM) tissues stained for microtubule-associated protein 2 (MAP2; white), amyloid-beta (Aβ; magenta), and 4’,6-diamidino-2-phenylindole (DAPI; cyan) at 24 hours, 2, 5, and 10 weeks post-injury. Note that silk autofluorescence is visible in the magenta and cyan channels. Scale bar: 100 μm. CCI: Controlled cortical impact.

***Additional Figure 5:*** *Treatment with low-concentration mitomycin-C does not permanently inhibit mitosis, but may slightly reduce regeneration.*

Additional Figure 5 Treatment with low-concentration mitomycin-C does not permanently inhibit mitosis, but may slightly reduce regeneration.
(A) Representative confocal images of Tuj1-stained triculture (NAM) tissues that were treated with 100 ng/mL mitomycin-C for 4 days prior to injury to mitotically inactivate cells. Scale bar: 100 μm. (B) Quantification of Tuj1^+^ network density in confocal z-stack images of mitomycin-treated NAM tissues after injury. (C) Confocal images of NAM tissues, with or without mitomycin treatment, stained for the mitotic marker Ki67 (magenta). Scale bar = 50 μm. Data presented as mean ± SD. *P < 0.05, ****P < 0.0001 (one-way analysis of variance with Dunnett’s T3 post hoc test) between Sham and CCI. CCI: controlled cortical impact.

***Additional Figure 6:*** *Long-term culture capability of 3D brain-like tissues.*

Additional Figure 6 Long-term culture capability of 3D brain-like tissues.
(A) Calcein-AM live cell staining of 13-month-old triculture (NAM) and neuron monoculture (N) tissues. Scale bars: 100 μm. (B) Immunostaining of fixed 13-month-old NAM and N tissues for Tuj1 and microtubule associated protein 2 (MAP2), with 4’,6-diamidino-2-phenylindole (DAPI) nuclear stain. Scale bar: 50 μm.

## Data Availability

*All relevant data are within the paper, additional files, and supplementary materials (original uncropped Western blot images) Supplementary Materials-S1_raw_images.*
