# Supplementary material for: Methods for a bioengineered 3D human brain-like tissue model of neuroregeneration after traumatic brain injury
Source: Neural Regen Res. 2025 May 6;21(8):3620–8. doi: 10.4103/NRR.NRR-D-24-00497 (PMC13452648; doi:10.4103/NRR.NRR-D-24-00497)

iNSC markers

NAM Experiment #1

NAM Experiment #2

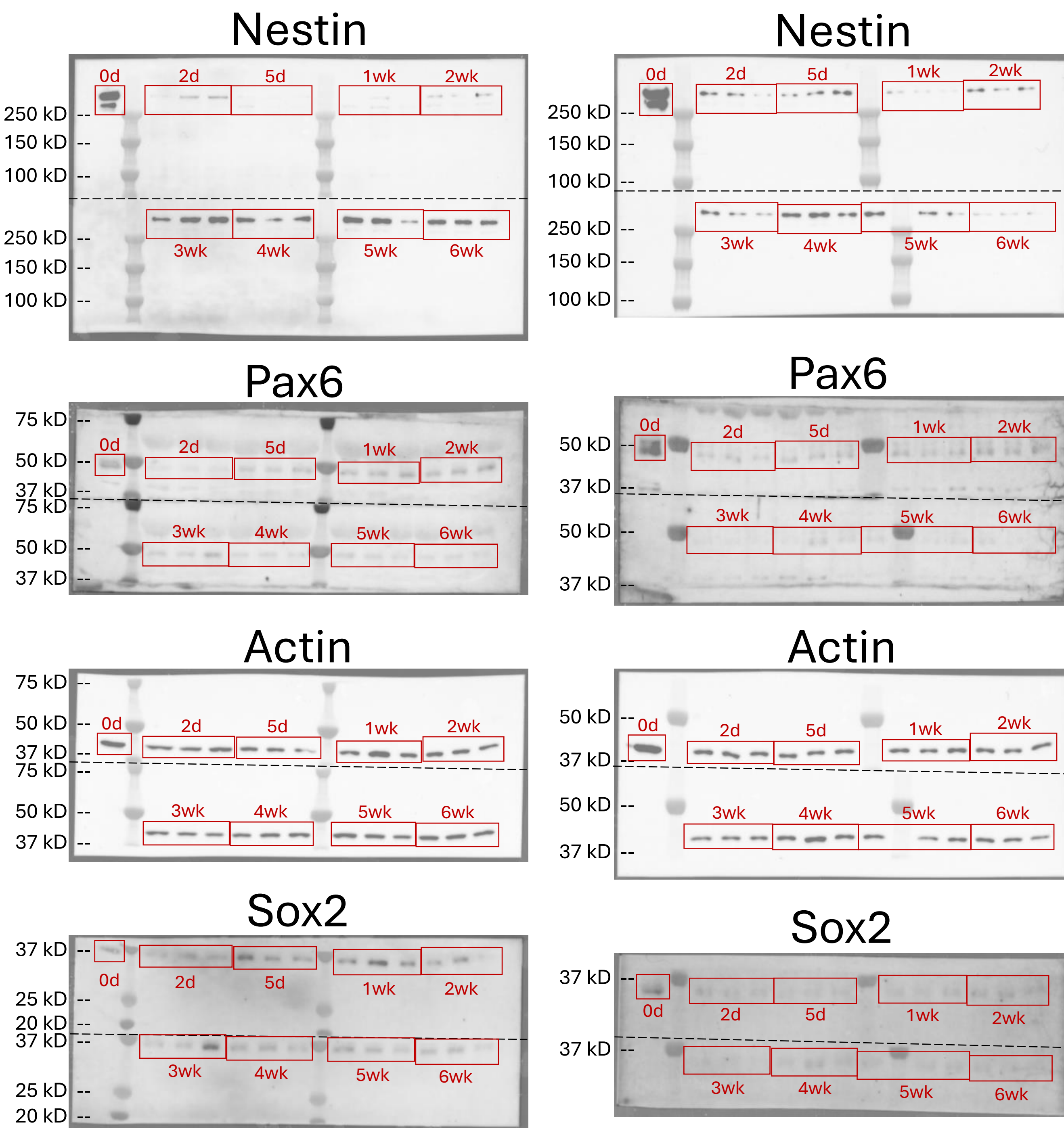

iNSC markers

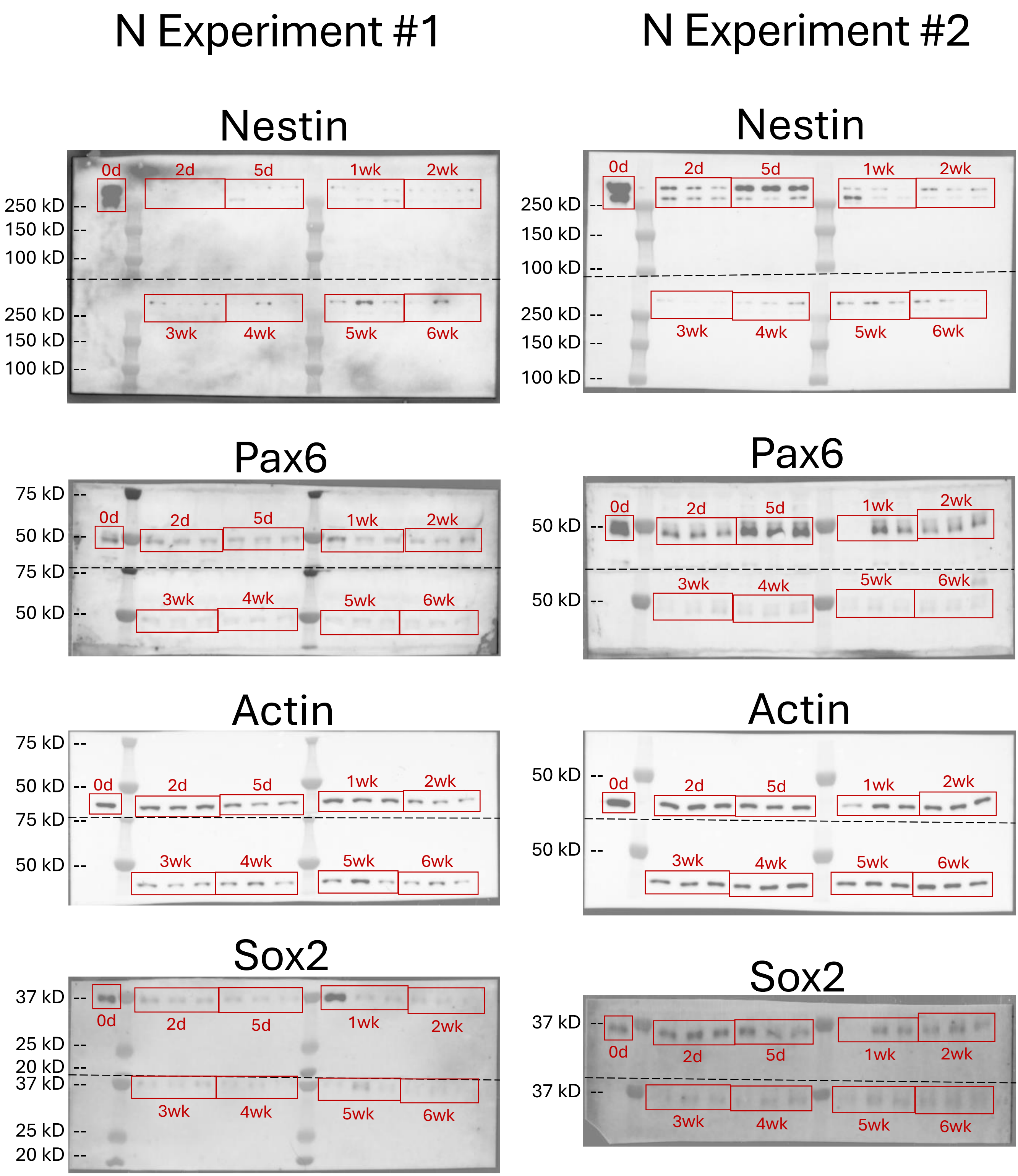

iNSC markers

A Experiment #1

Nestin

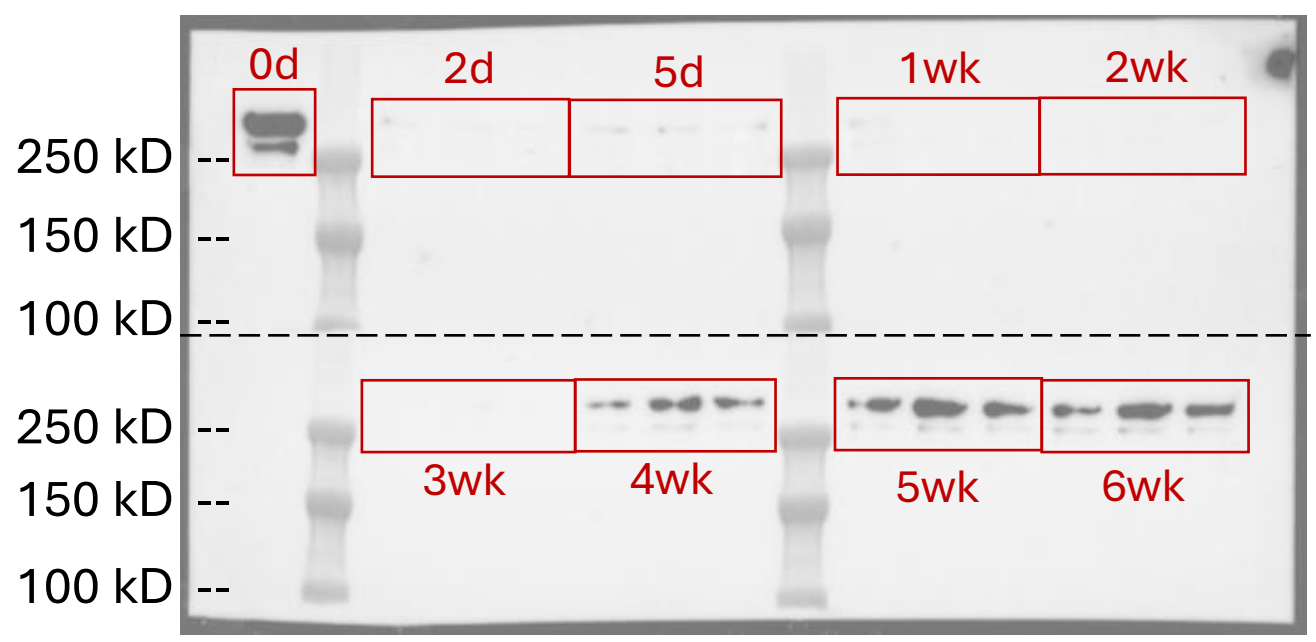

Pax6

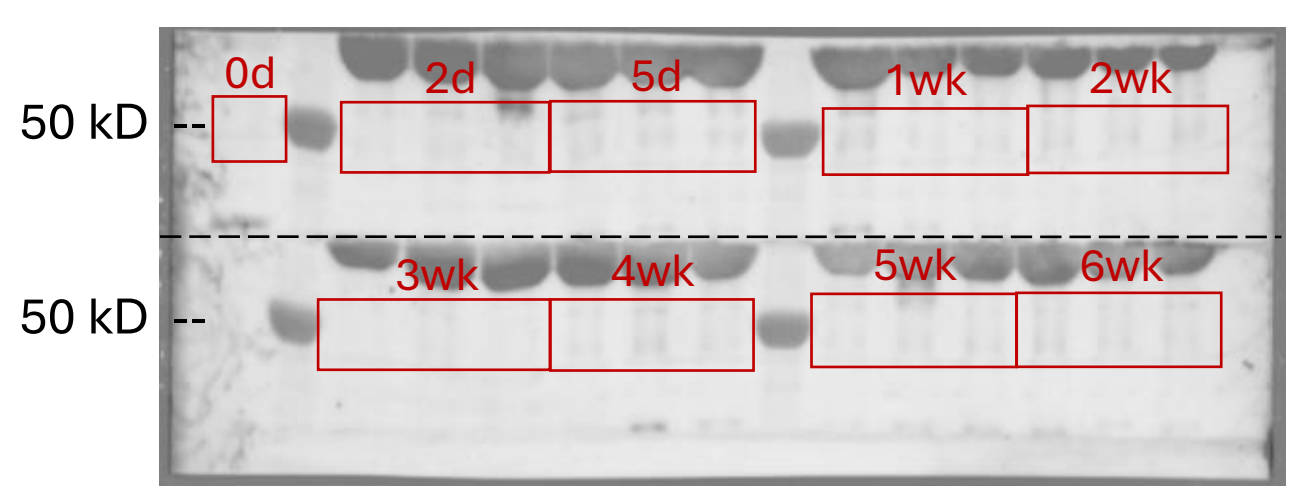

Actin

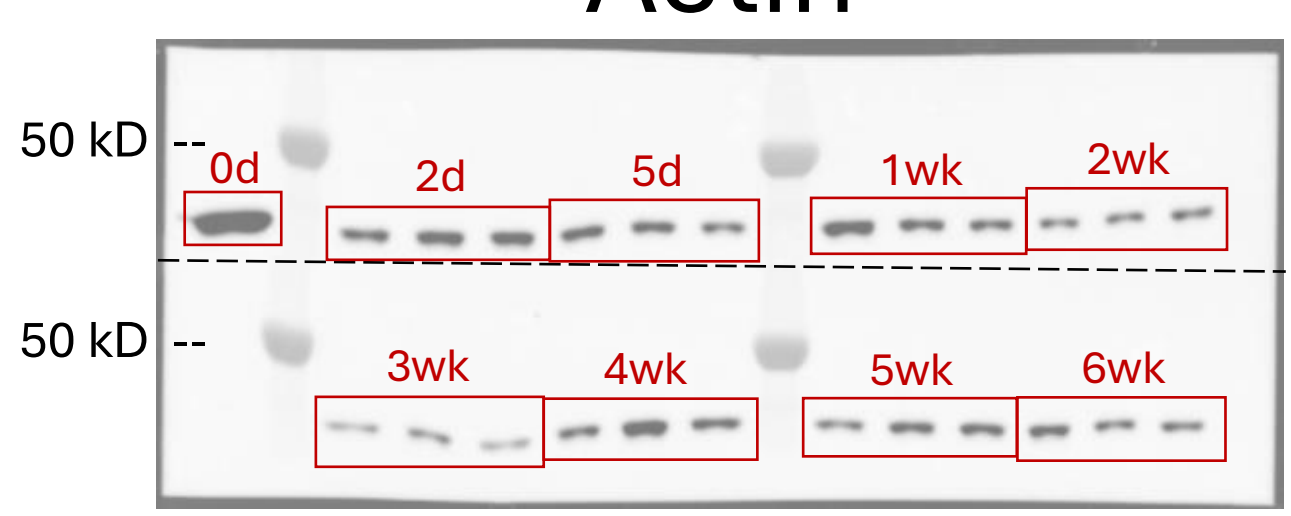

Sox2

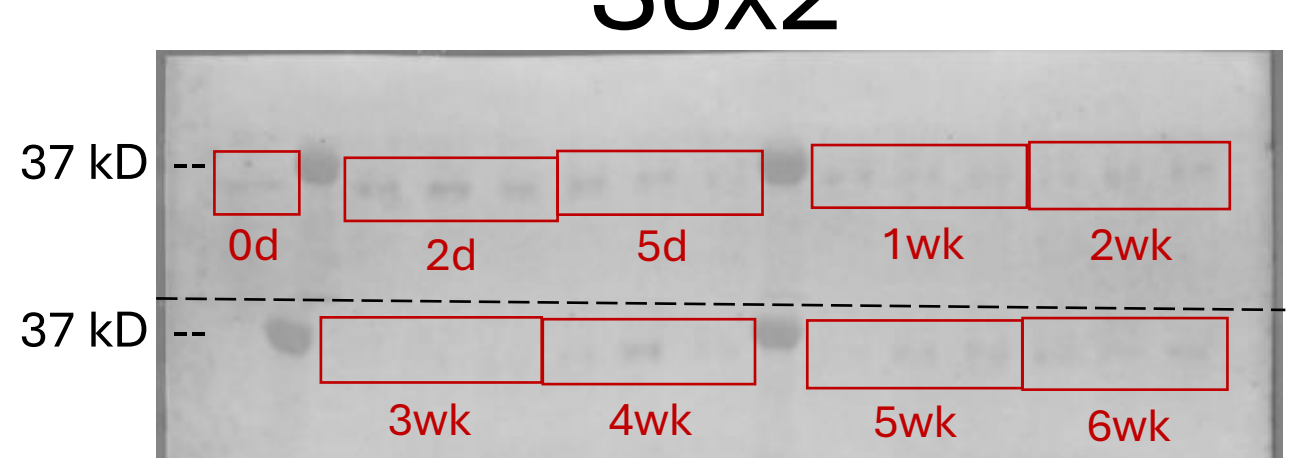

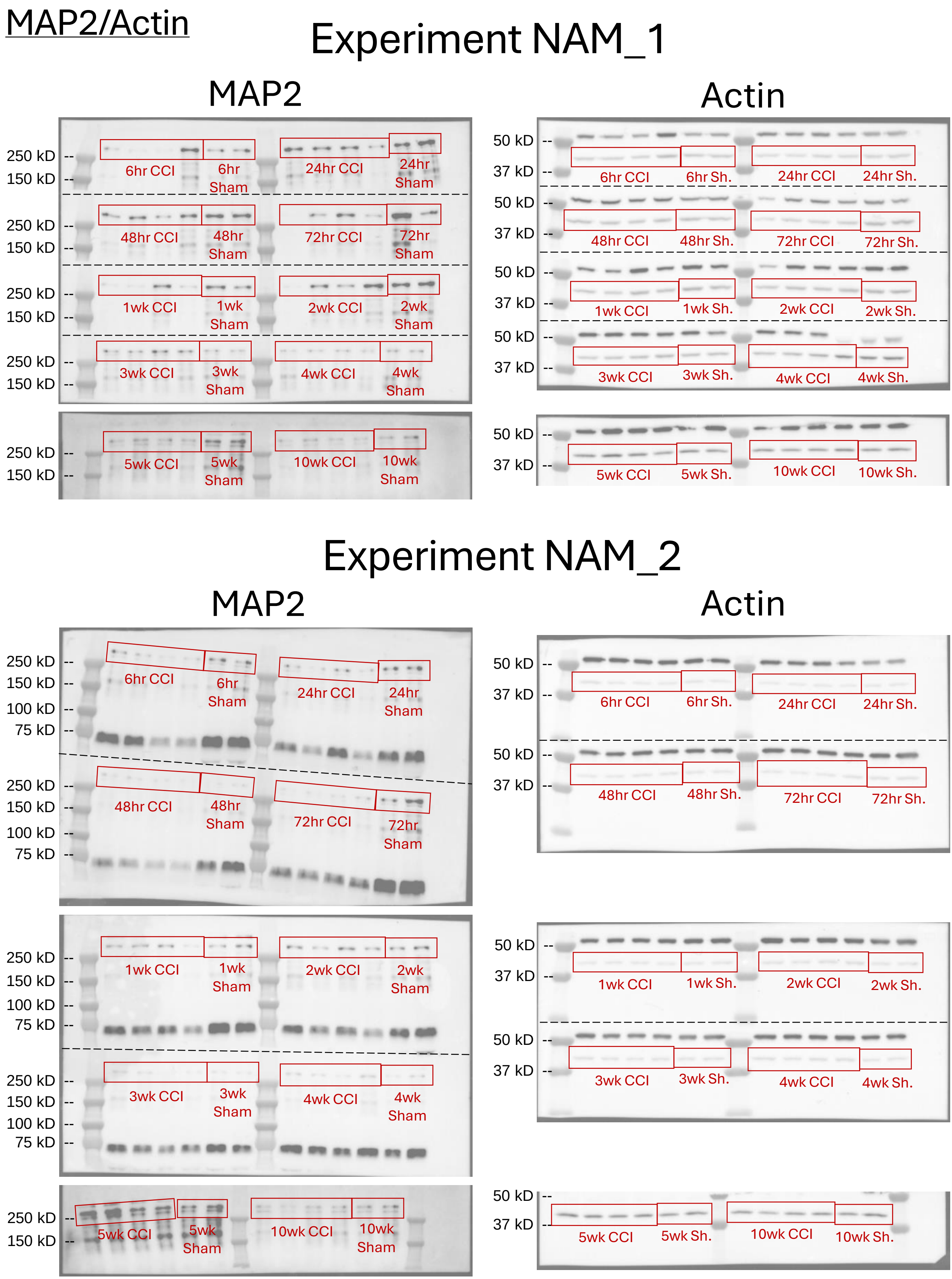
MAP2/Actin
Experiment NAM_1
MAP2
Actin
250 kD
150 kD
50 kD
37 kD
6hr CCI
6hr Sham
24hr CCI
24hr Sham
48hr CCI
48hr Sham
72hr CCI
72hr Sham
1wk CCI
1wk Sham
2wk CCI
2wk Sham
3wk CCI
3wk Sham
4wk CCI
4wk Sham
5wk CCI
5wk Sham
10wk CCI
10wk Sham
6hr Sh.
24hr Sh.
48hr Sh.
72hr Sh.
1wk Sh.
2wk Sh.
3wk Sh.
4wk Sh.
5wk Sh.
10wk Sh.
Experiment NAM_2
MAP2
Actin
250 kD
150 kD
100 kD
75 kD
50 kD
37 kD

<u>MAP2/Actin</u>

Experiment NAM_3

MAP2

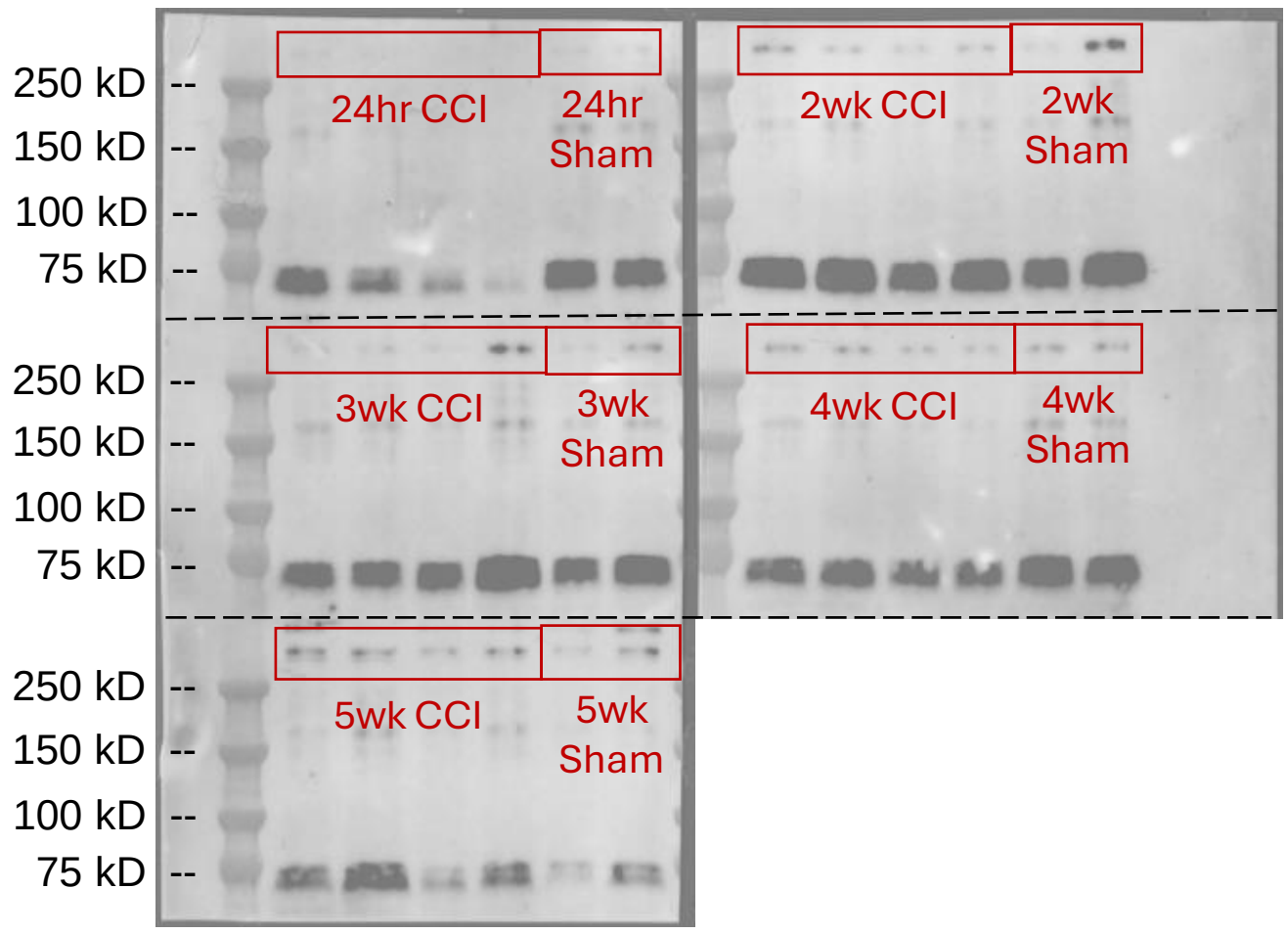

Actin

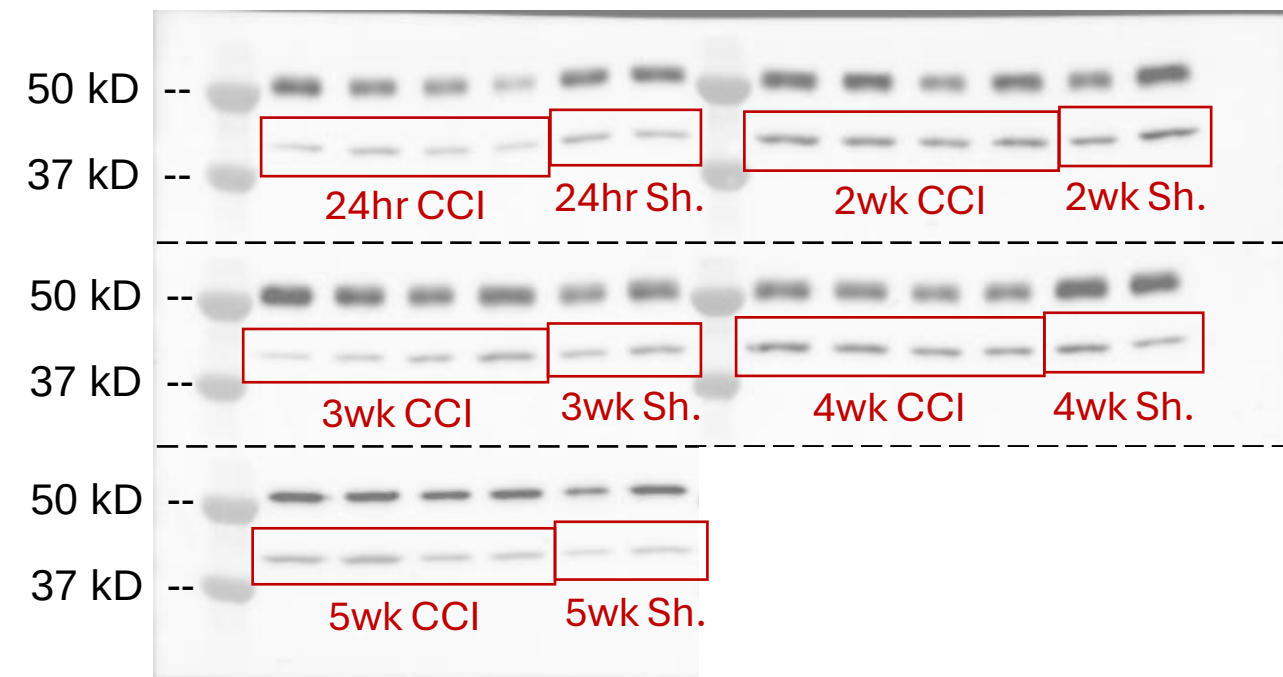

Experiment NAM_4

MAP2

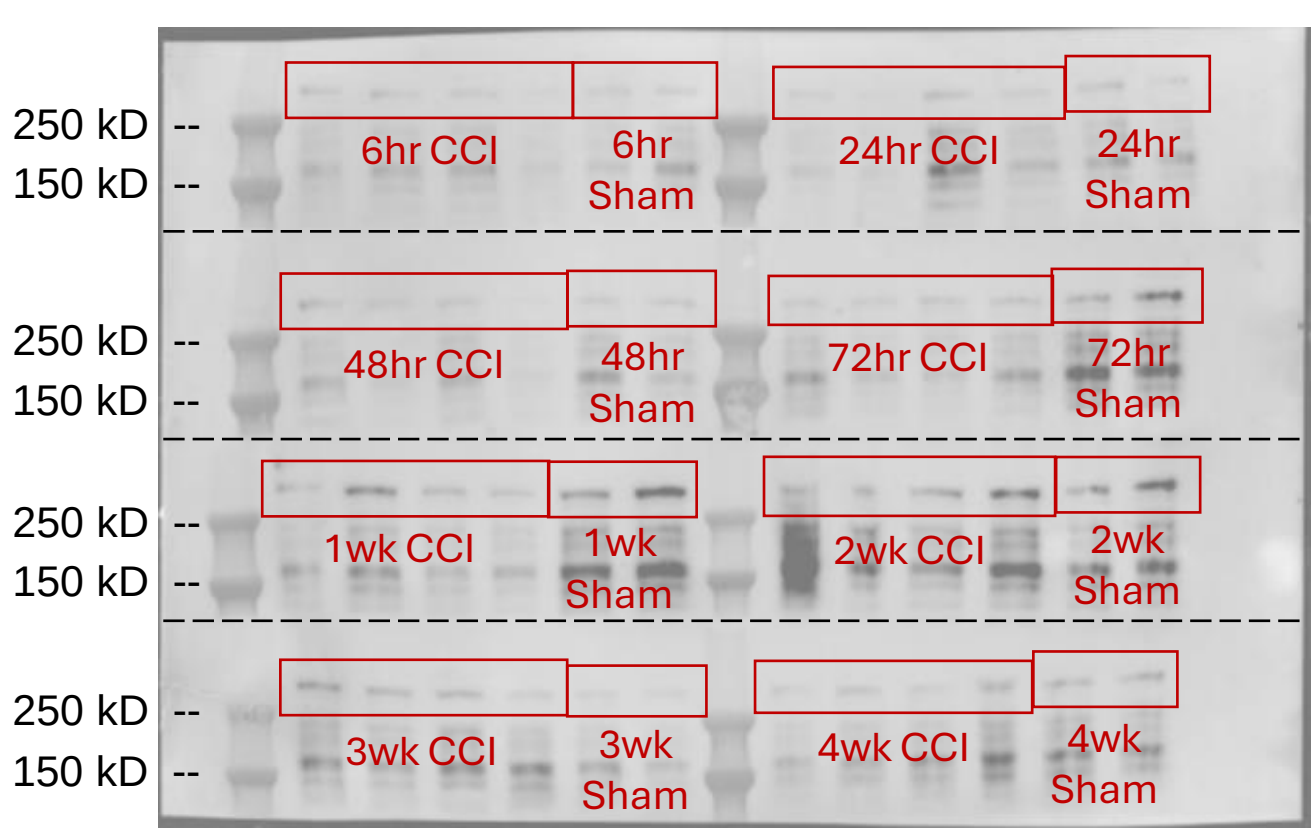

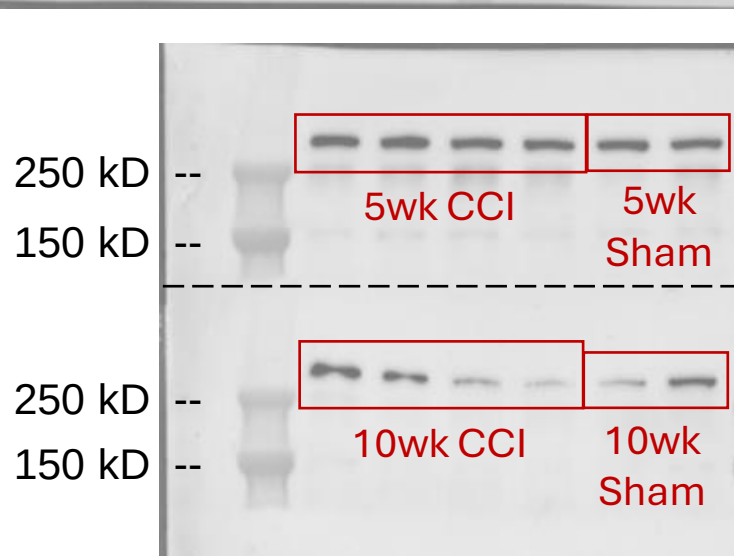

Actin

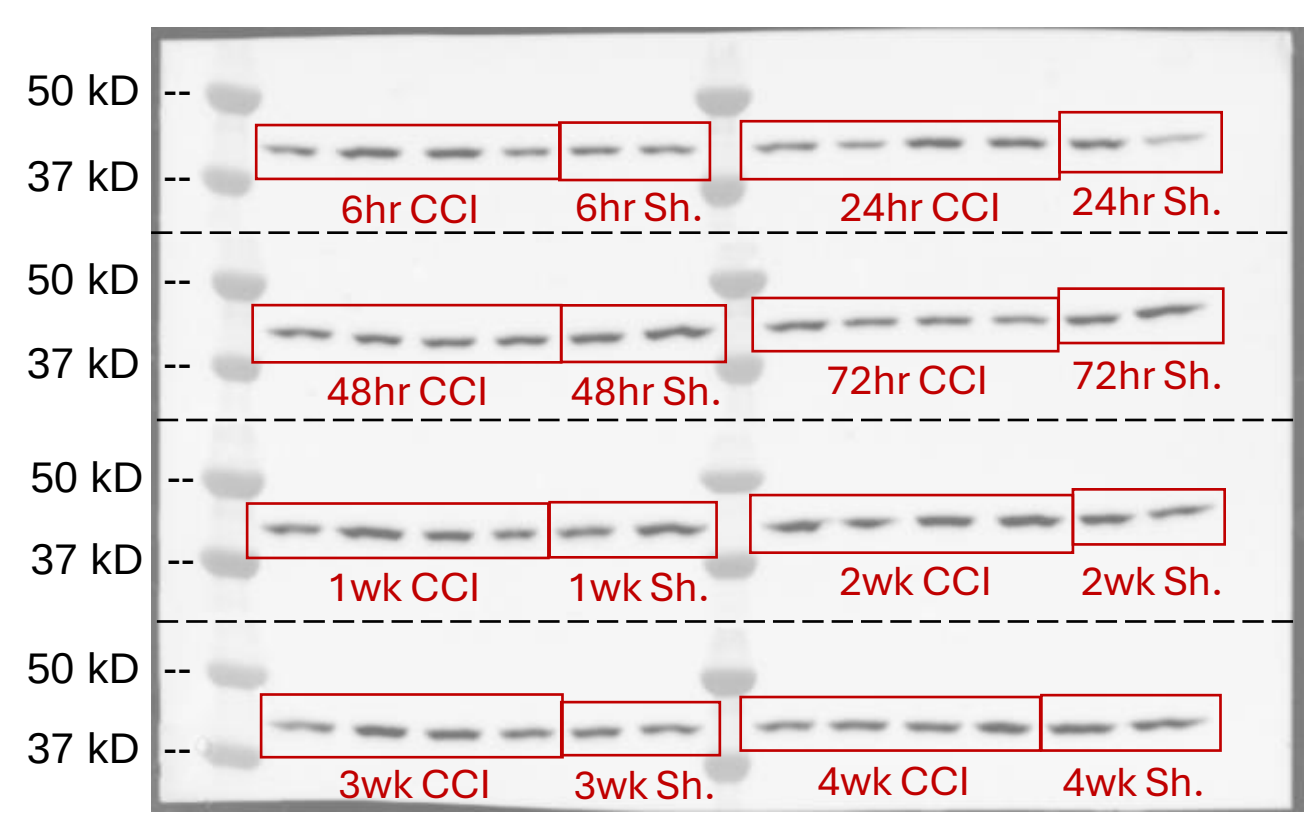

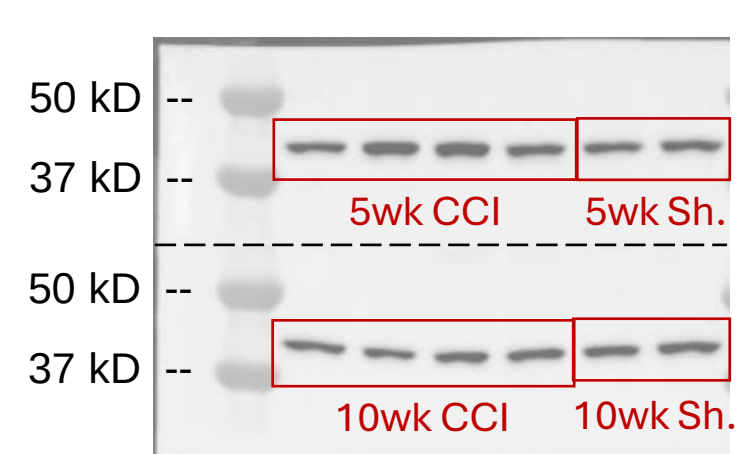

MAP2/Actin

Experiment N_1

MAP2

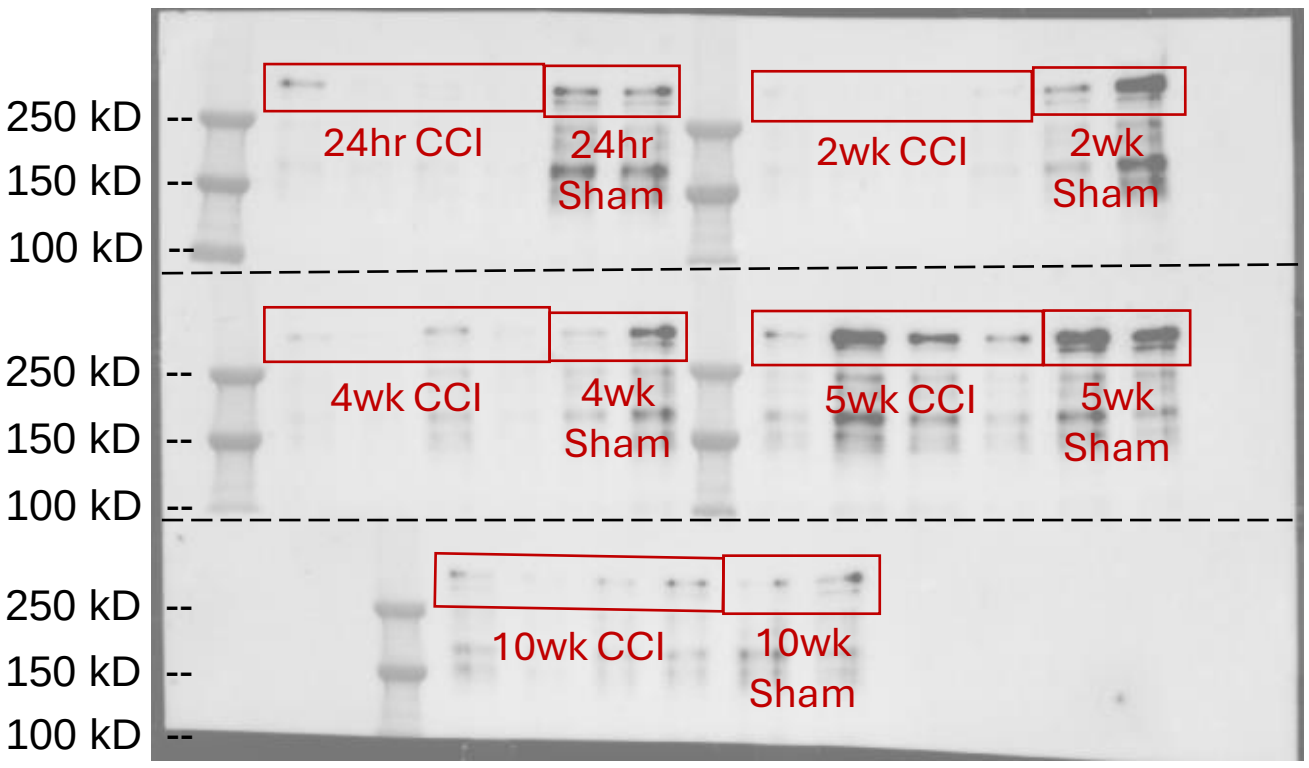

Actin

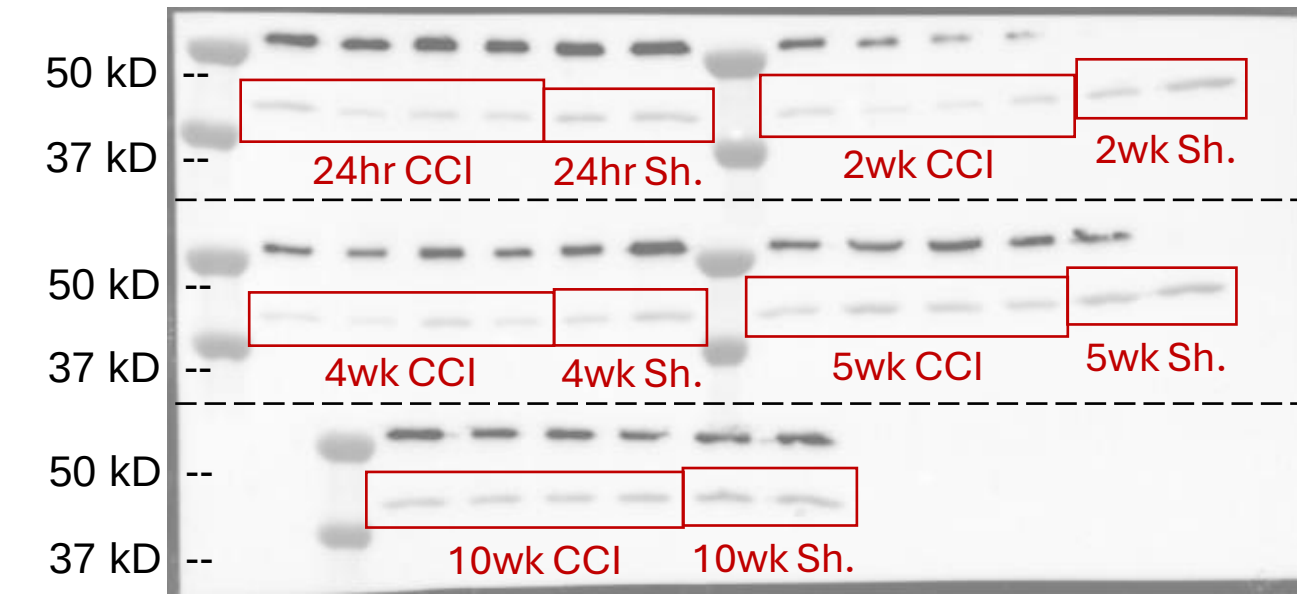

Experiment N_2

MAP2

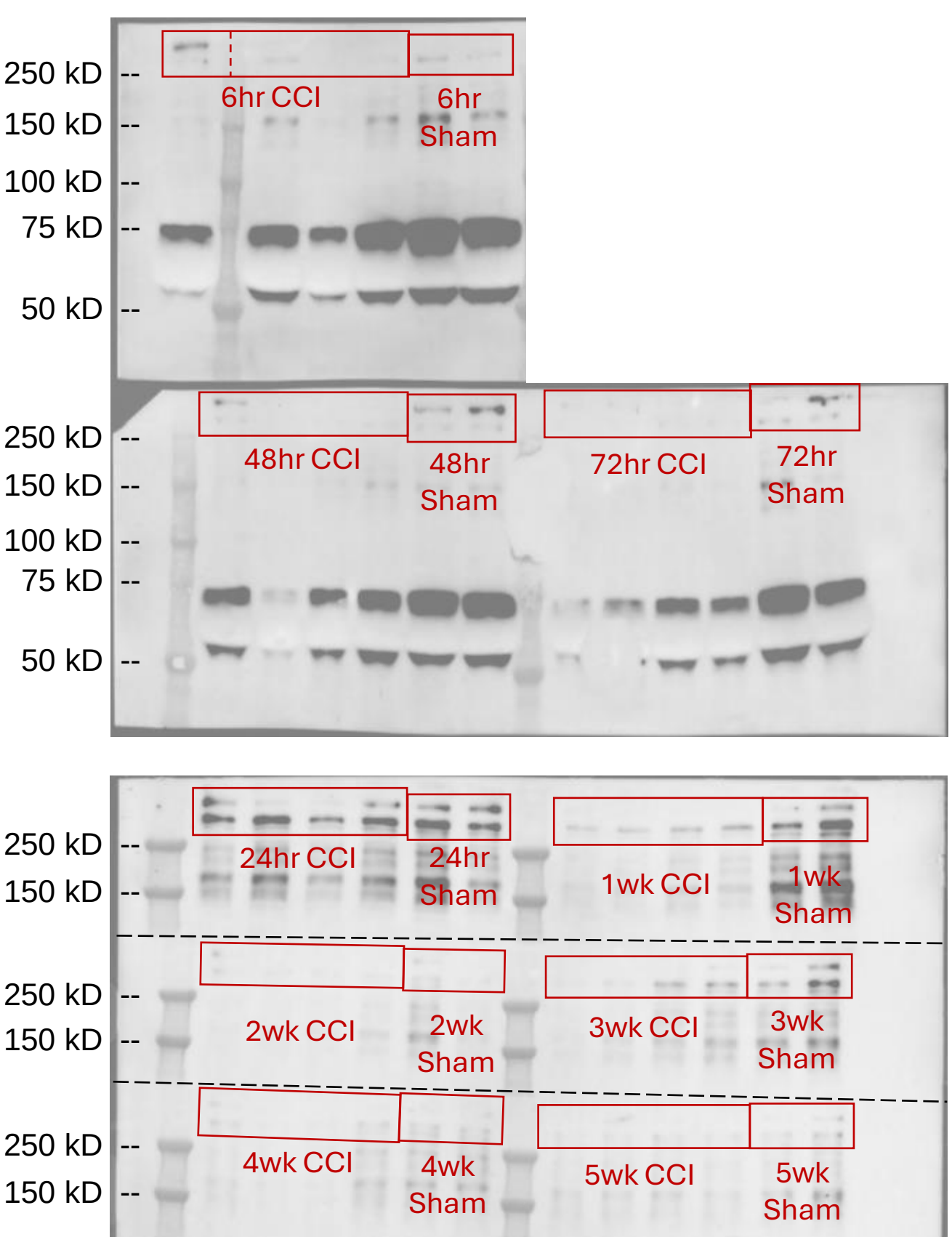

Actin

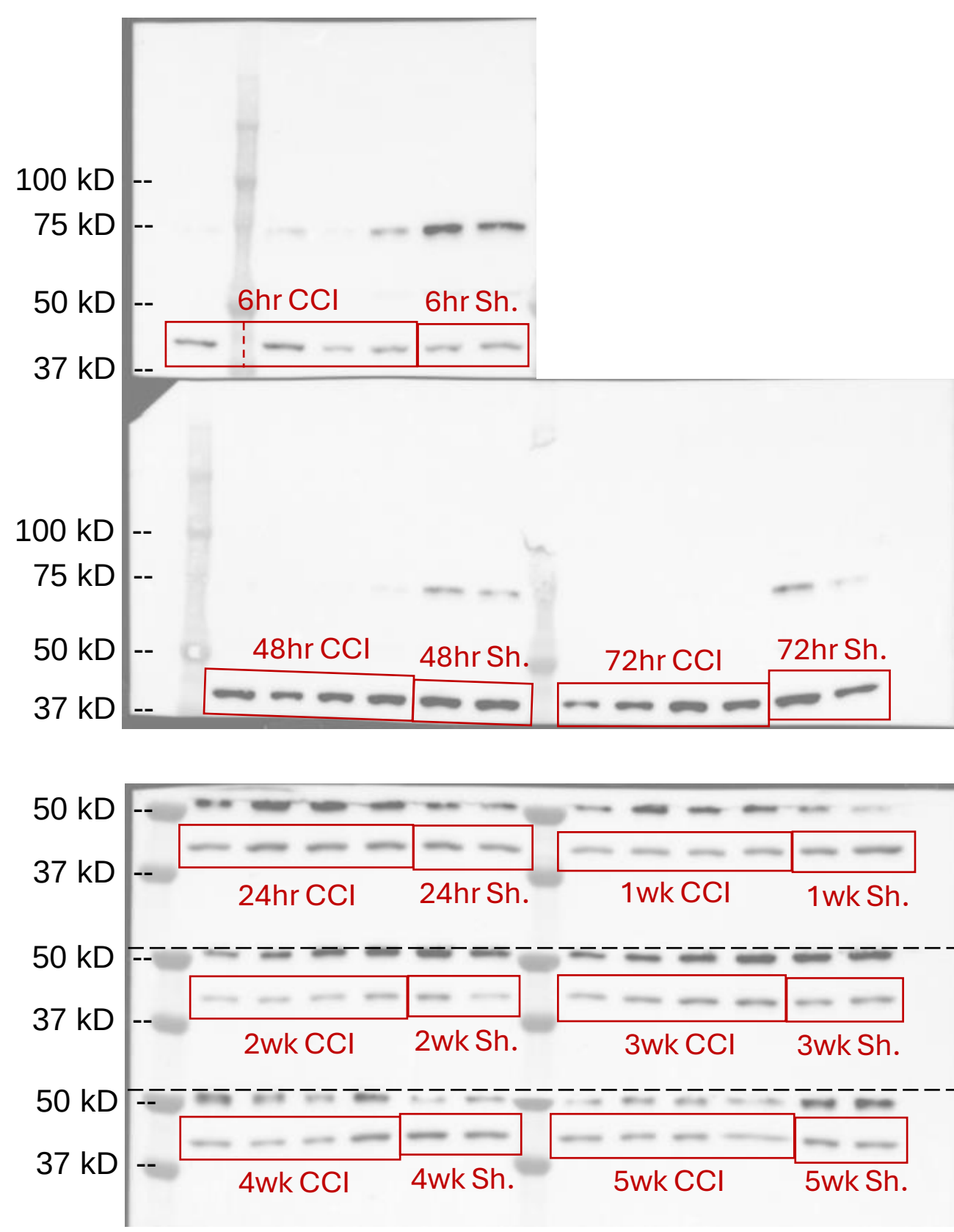

MAP2/Actin

Experiment N_3

MAP2

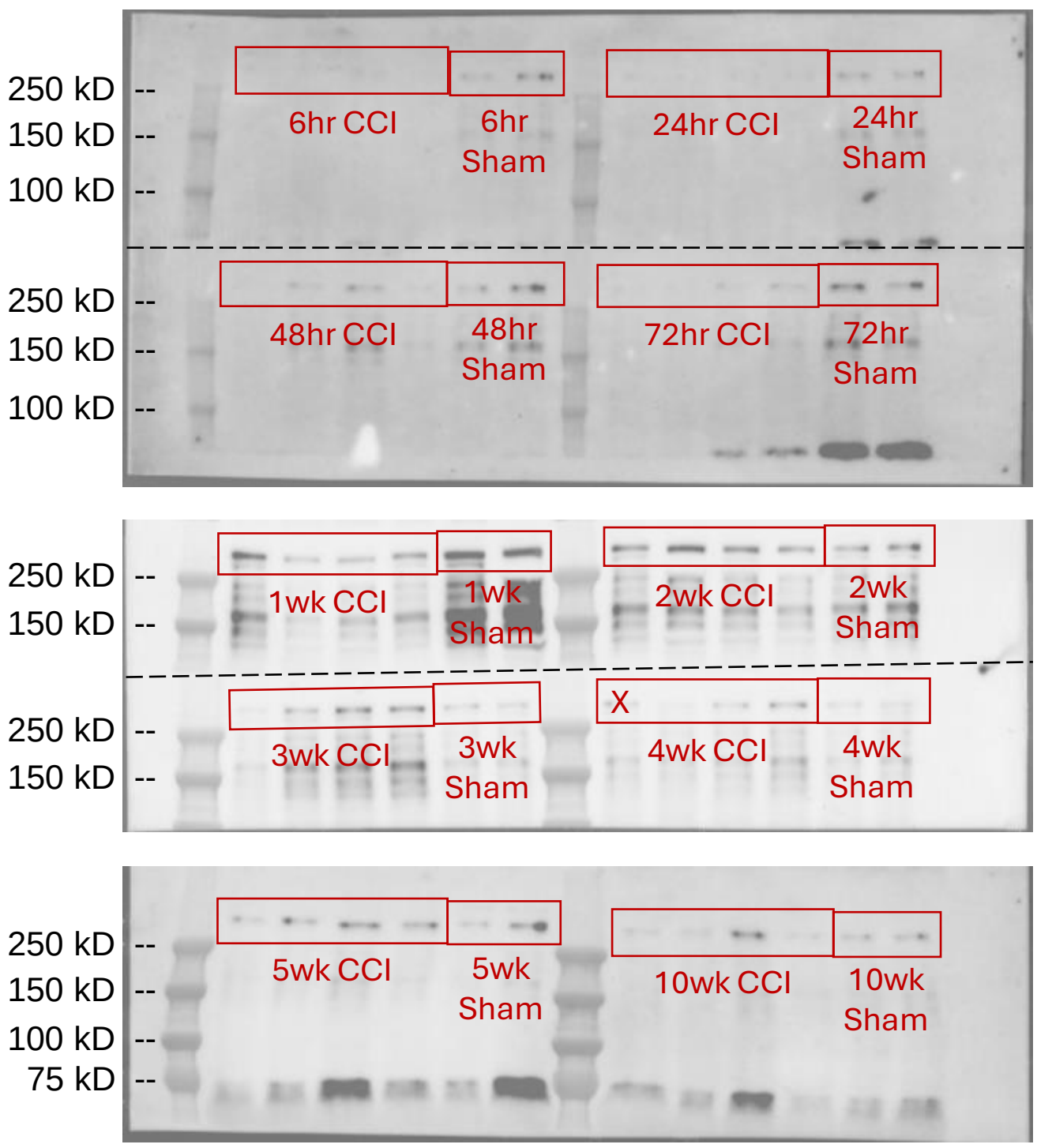

Actin

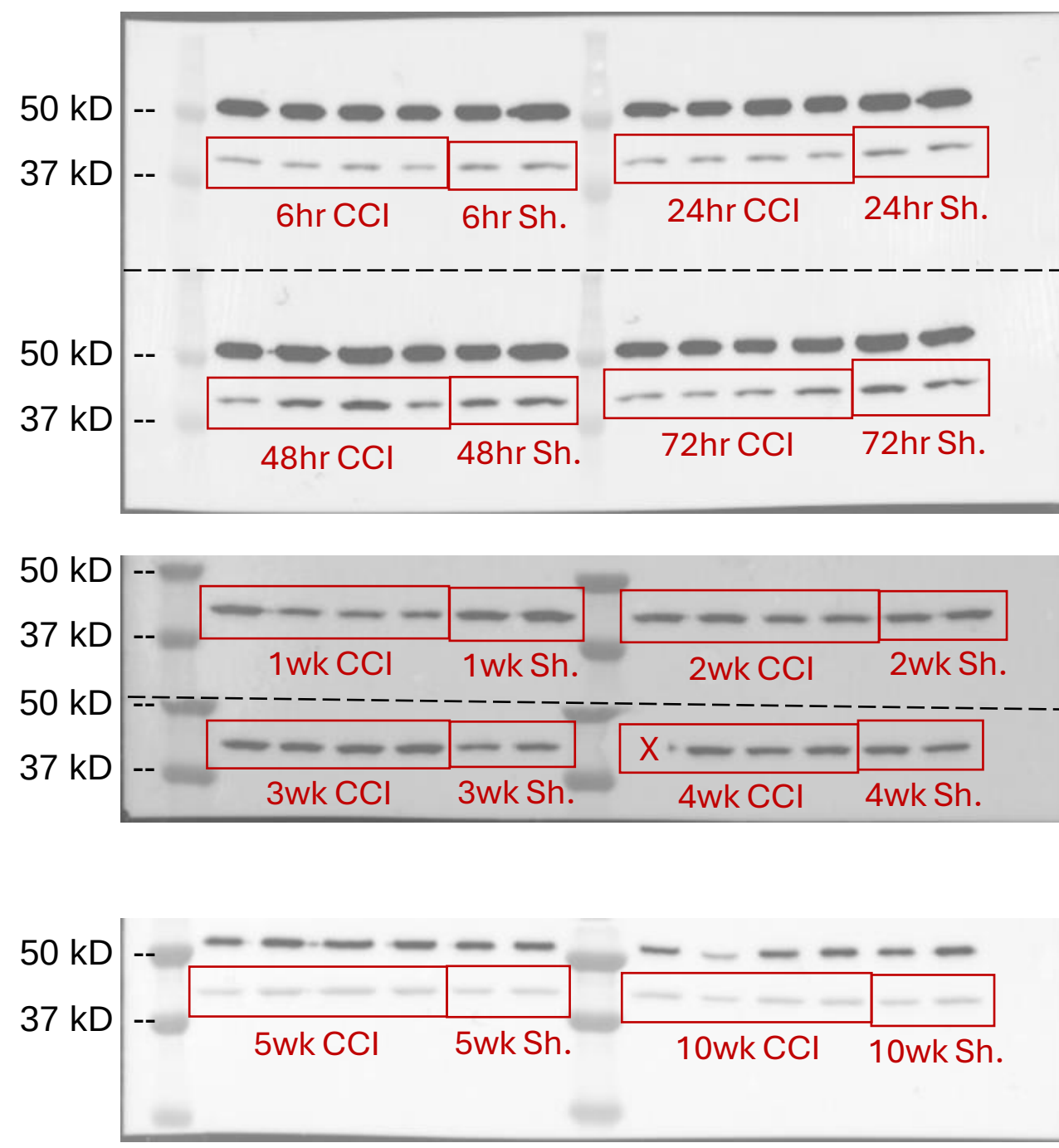

GAD65+67/NMDAR1

Experiment NAM_1

NMDAR1

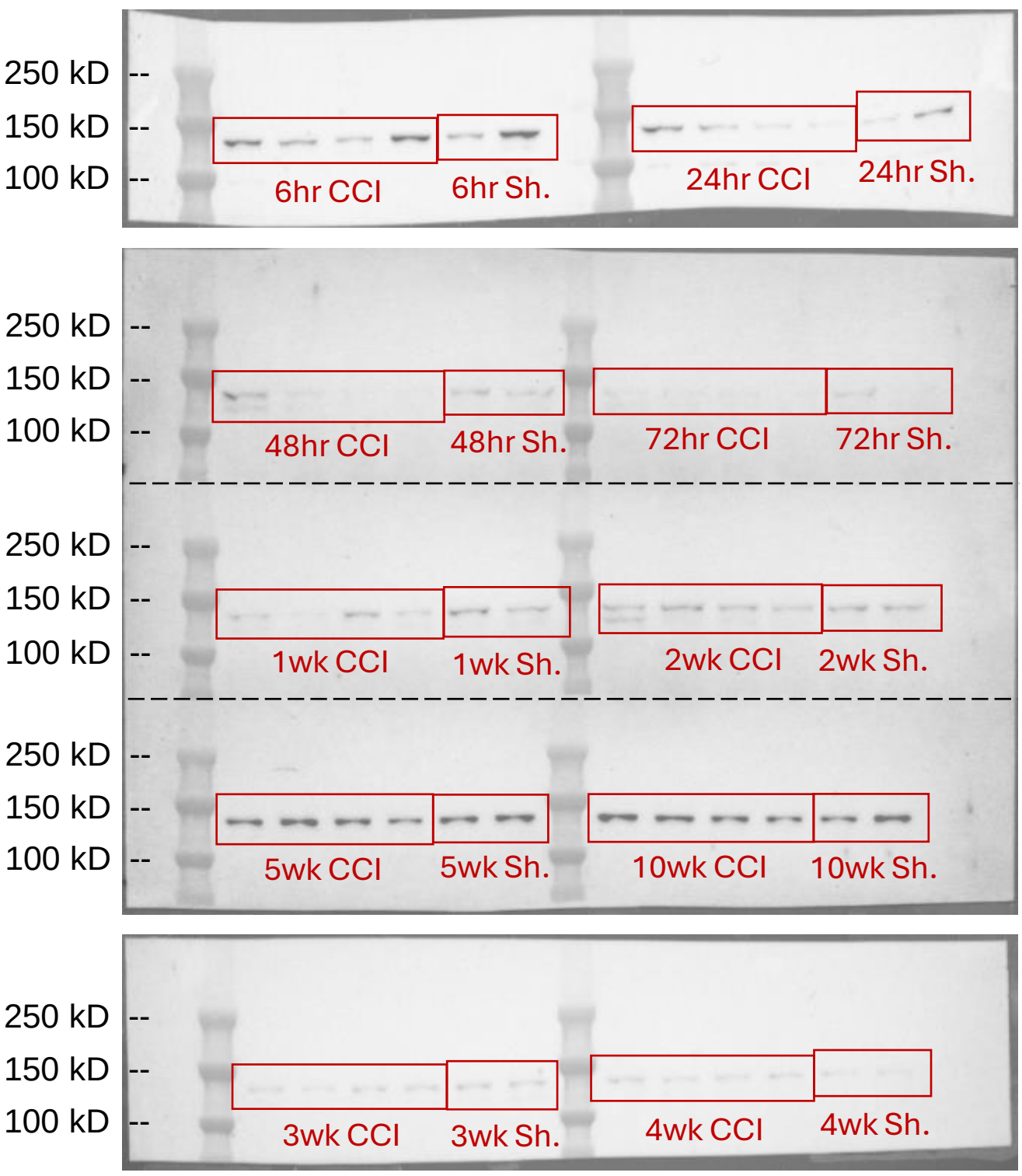

Tuj1

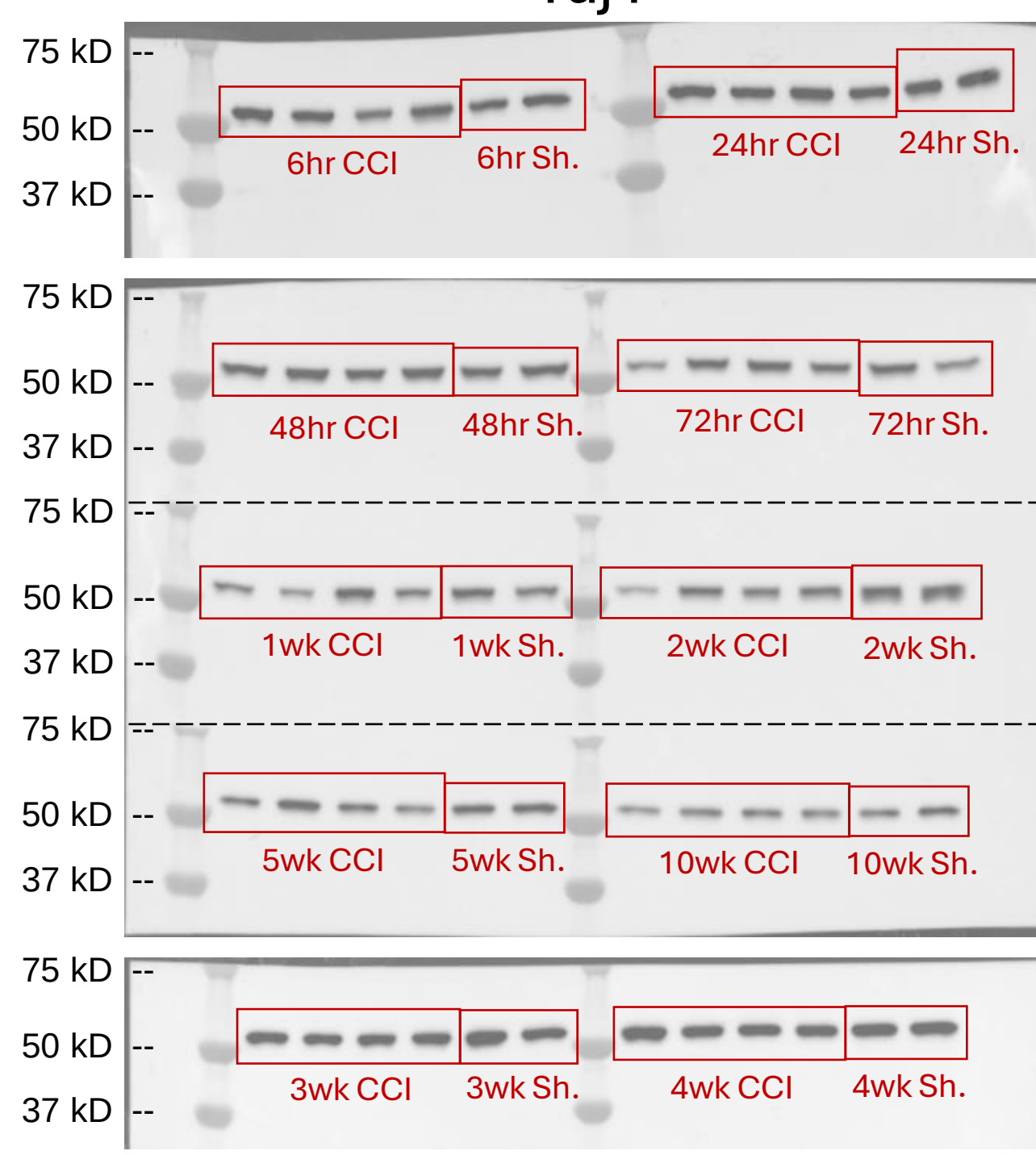

GAD65+67

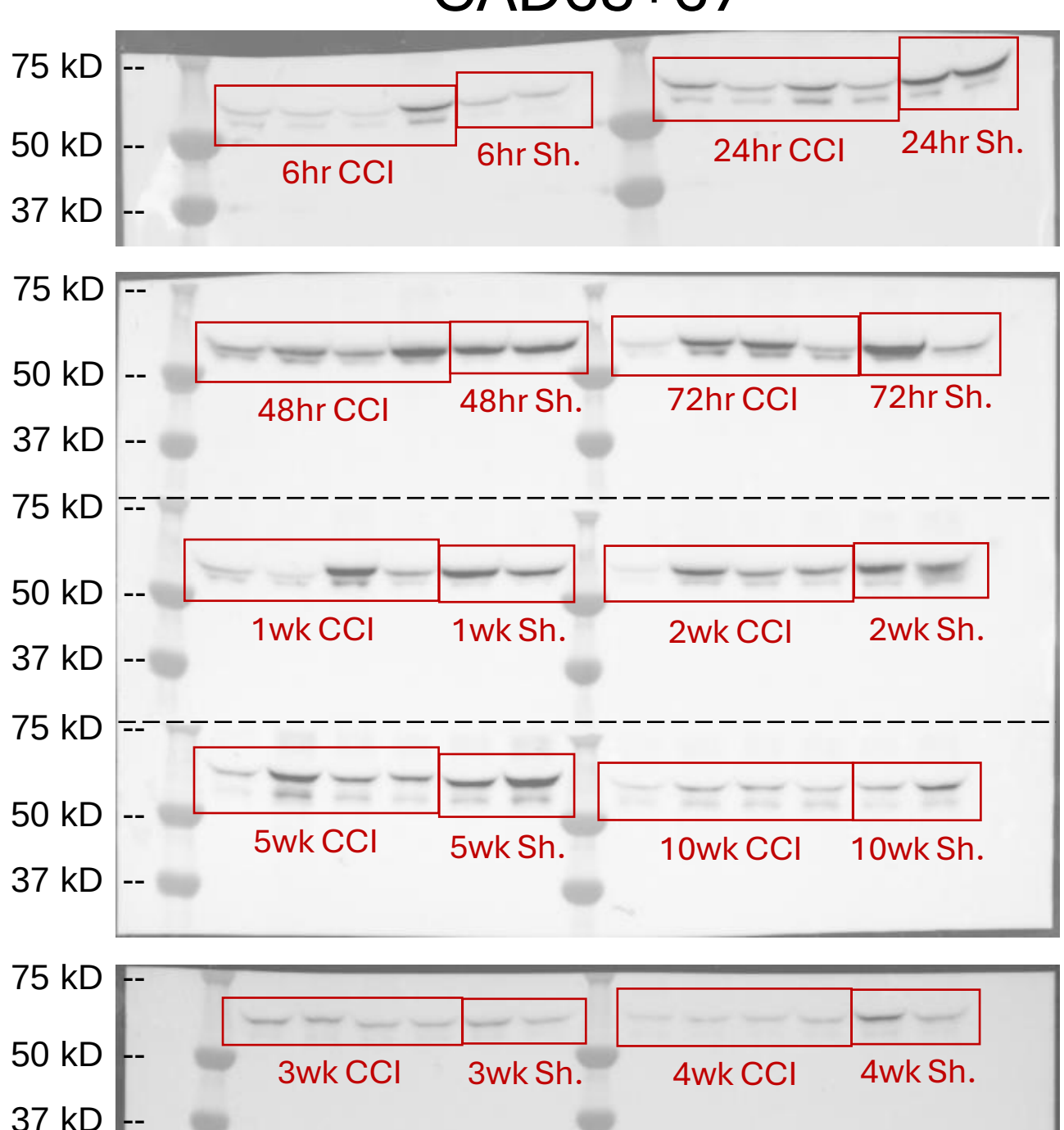

GAD65+67/NMDAR1

Experiment NAM_2

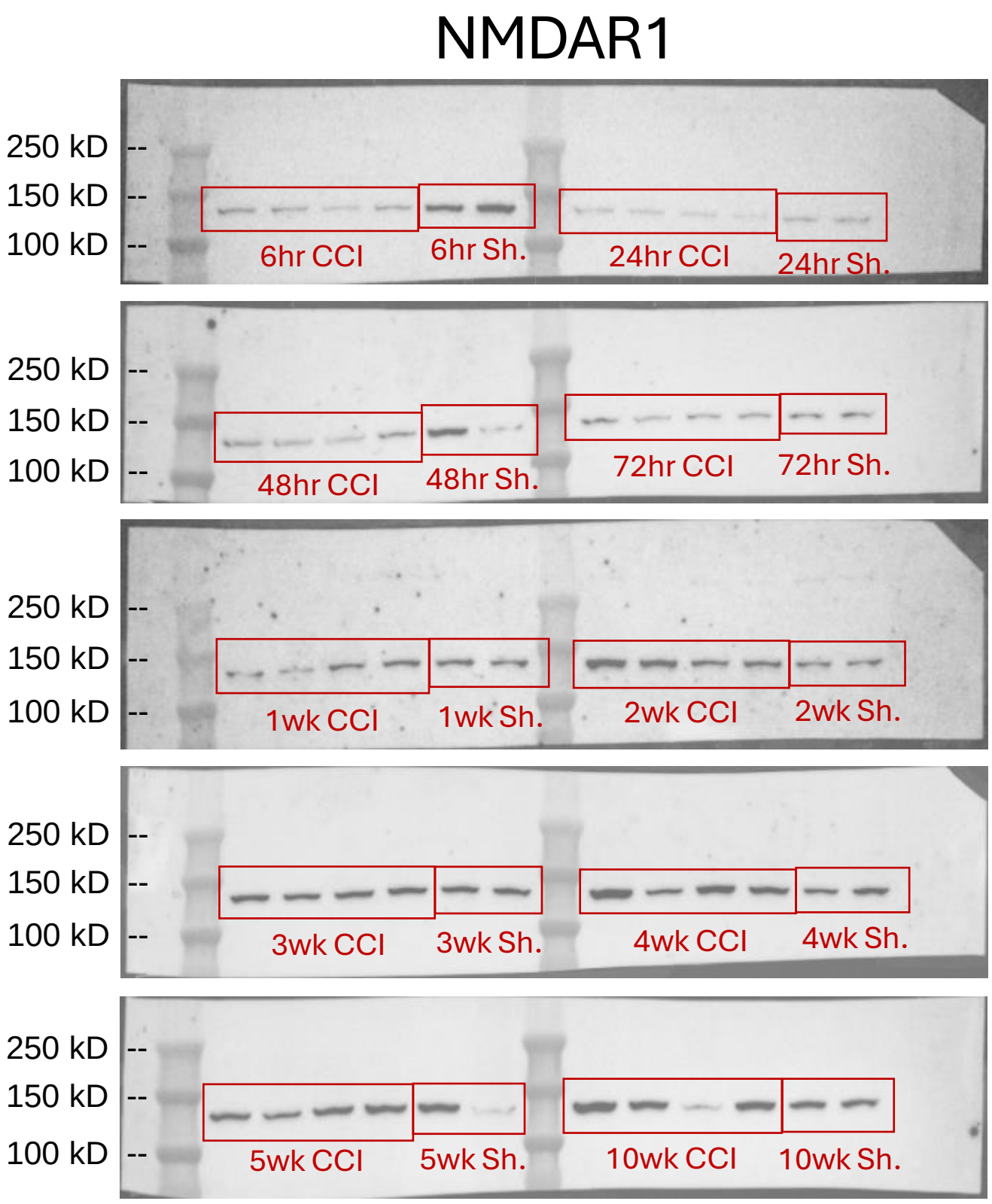

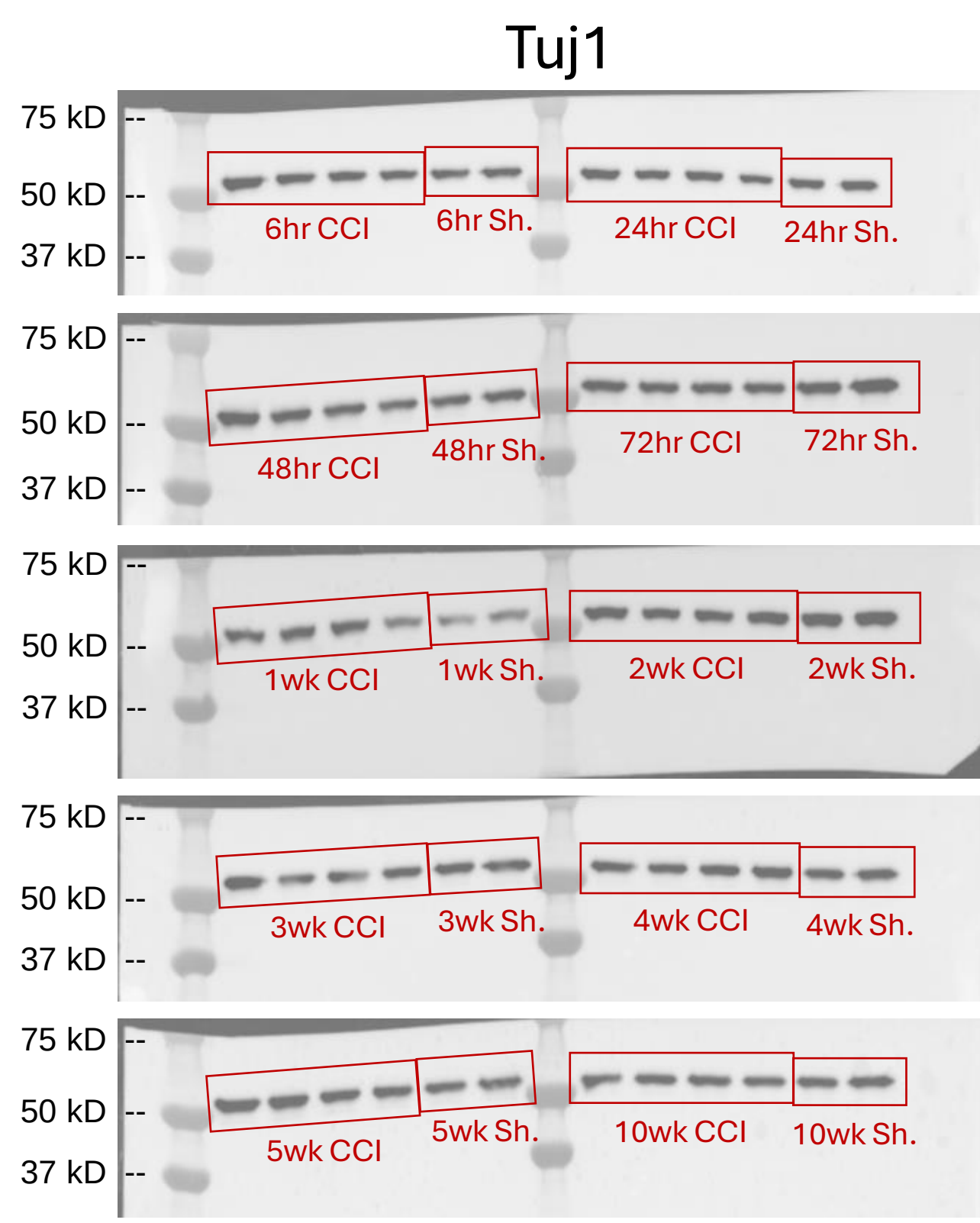

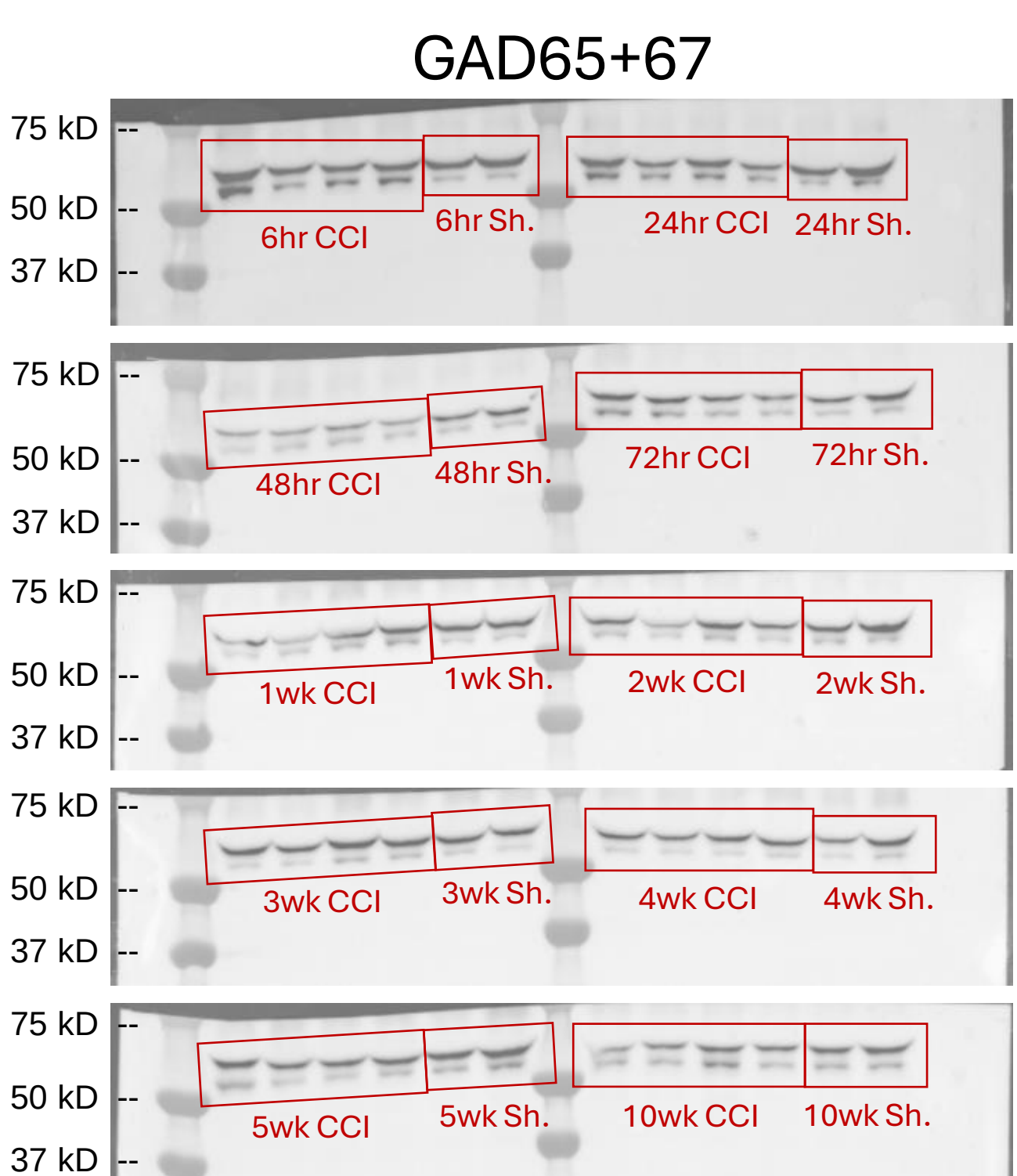

GAD65+67/NMDAR1

Experiment NAM_3

NMDAR1

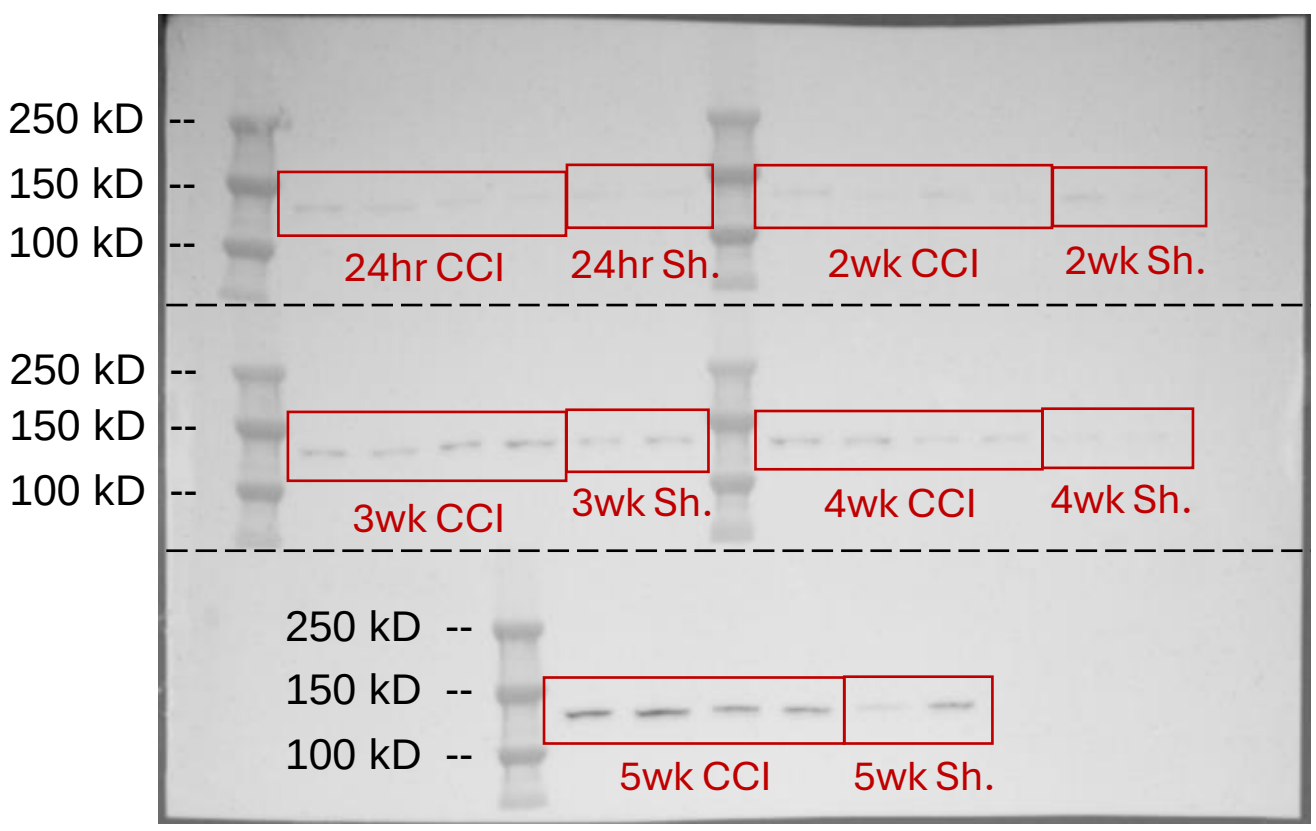

Tuj1

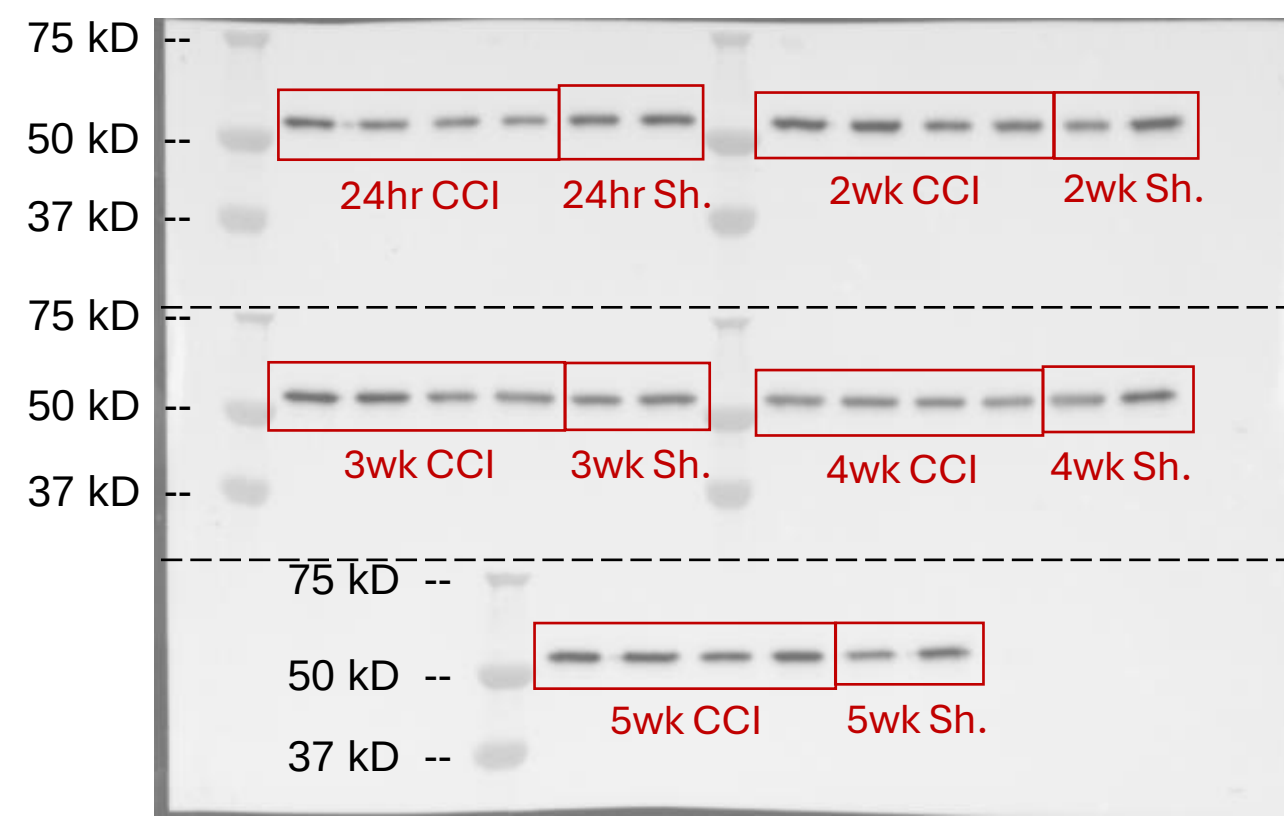

GAD65+67

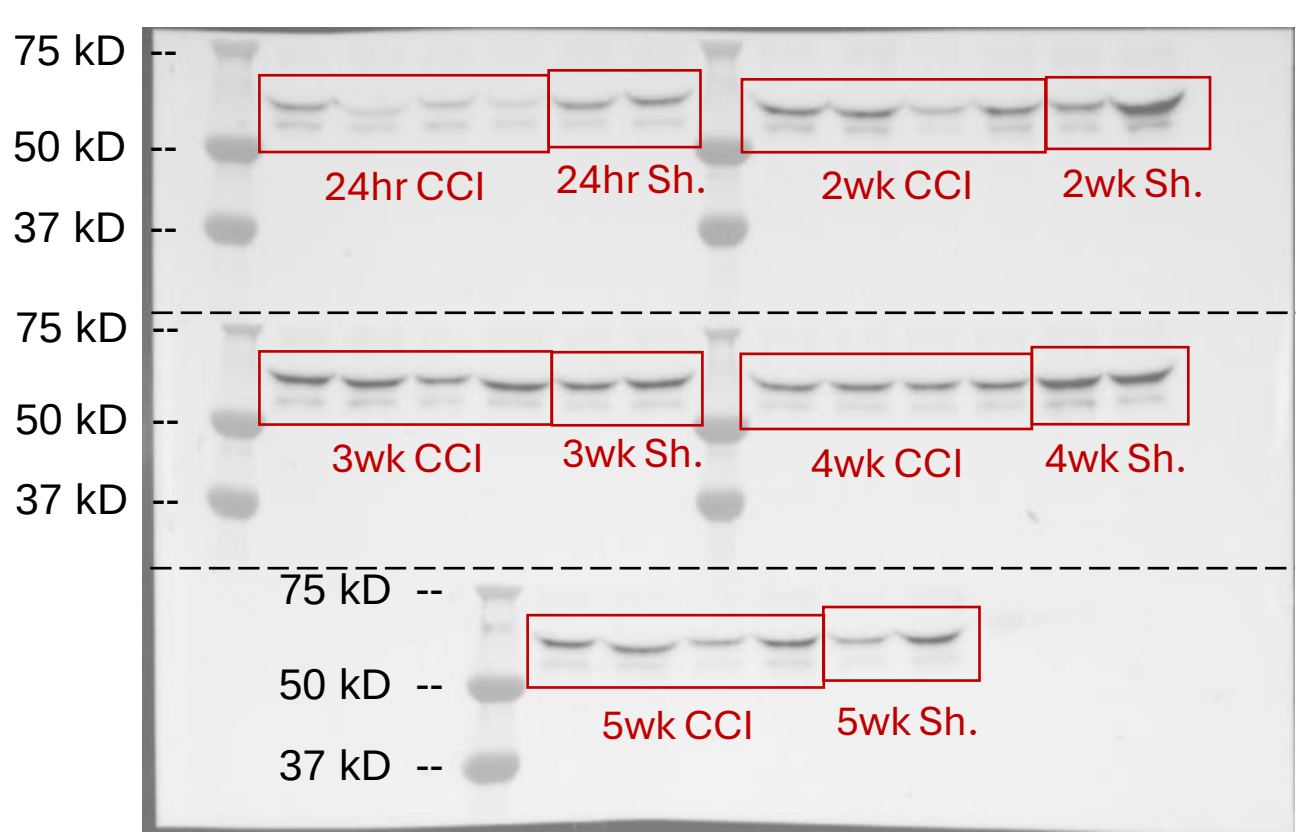

GAD65+67/NMDAR1

Experiment NAM_4

NMDAR1

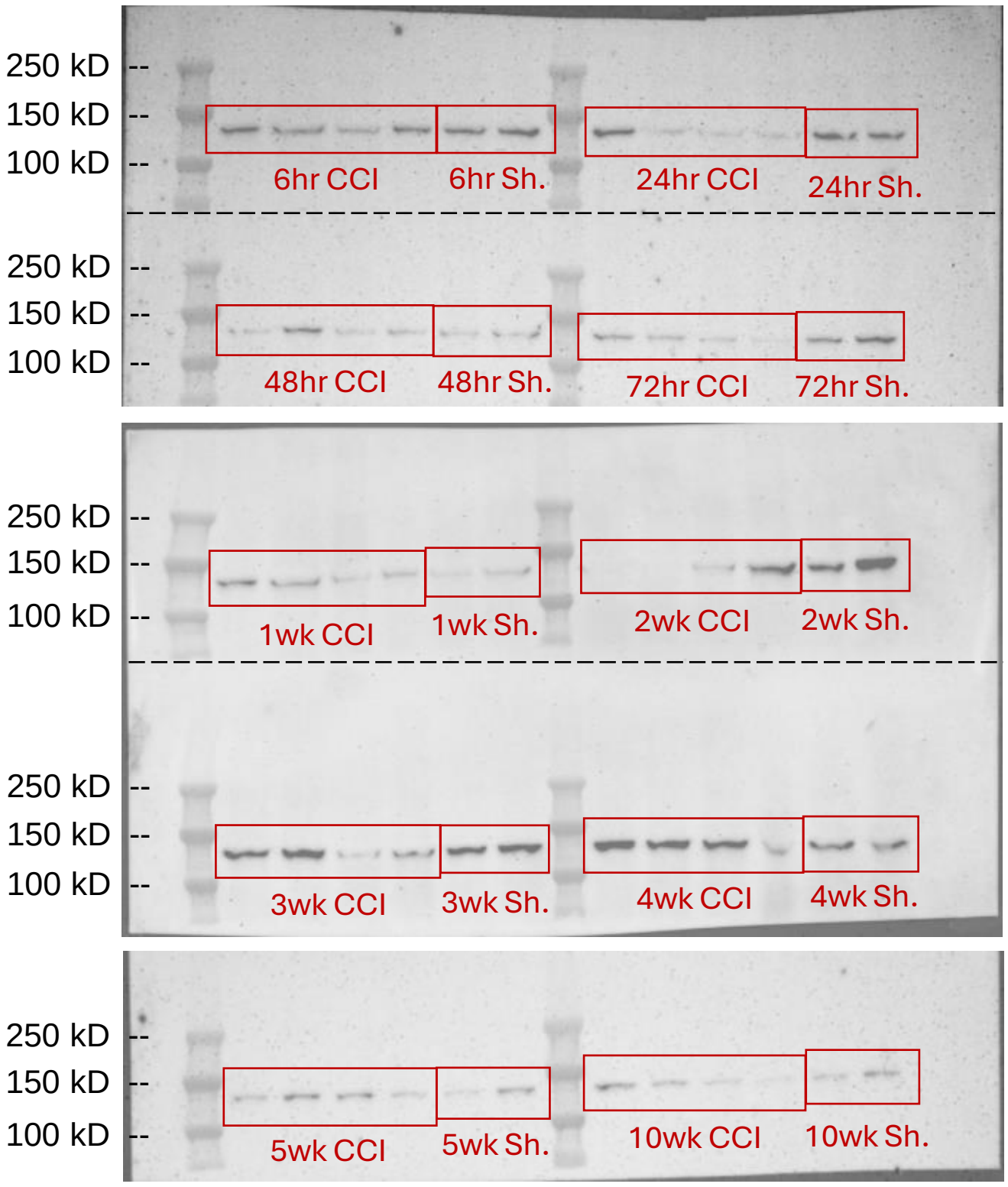

GAD65+67

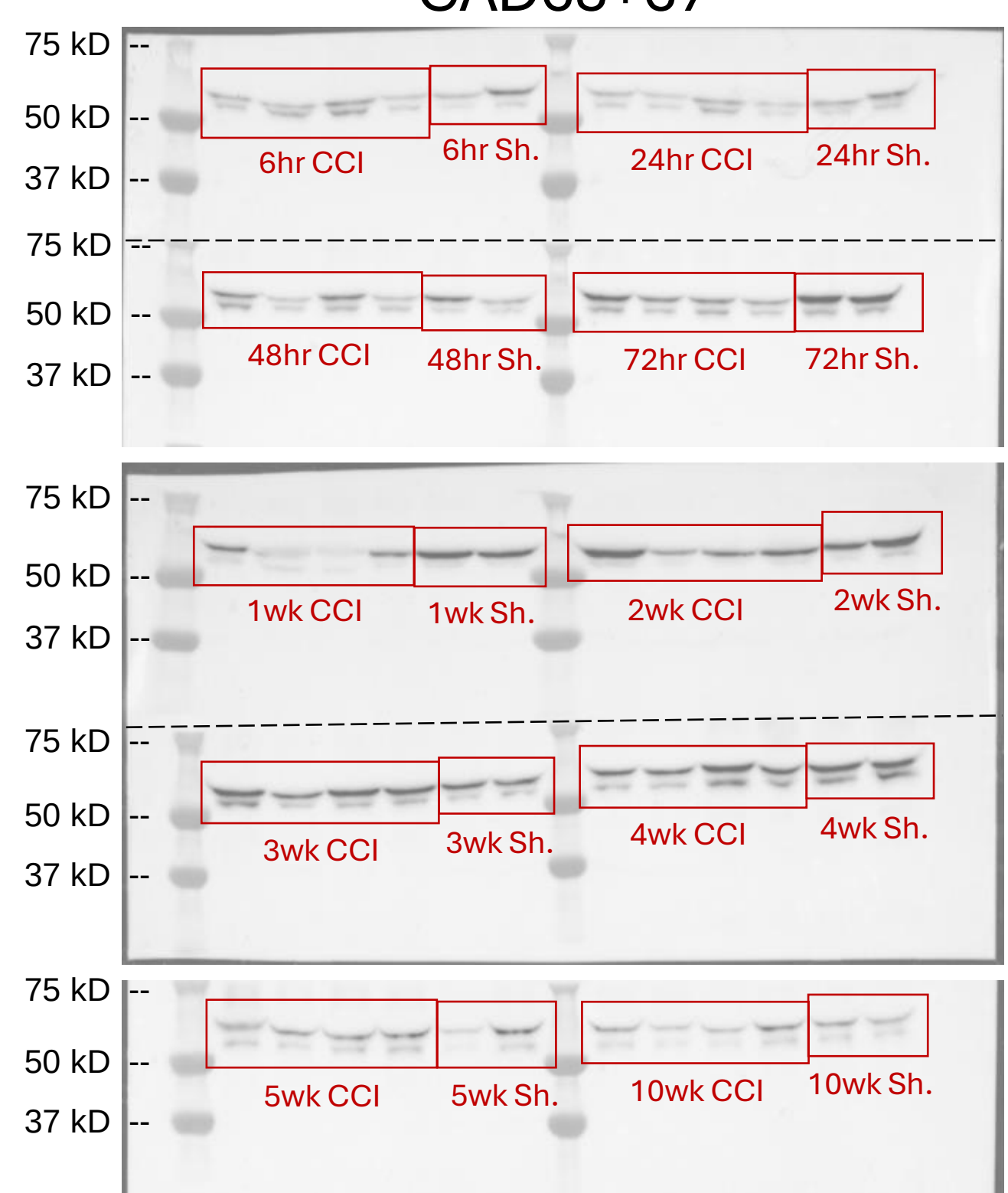

Tuj1

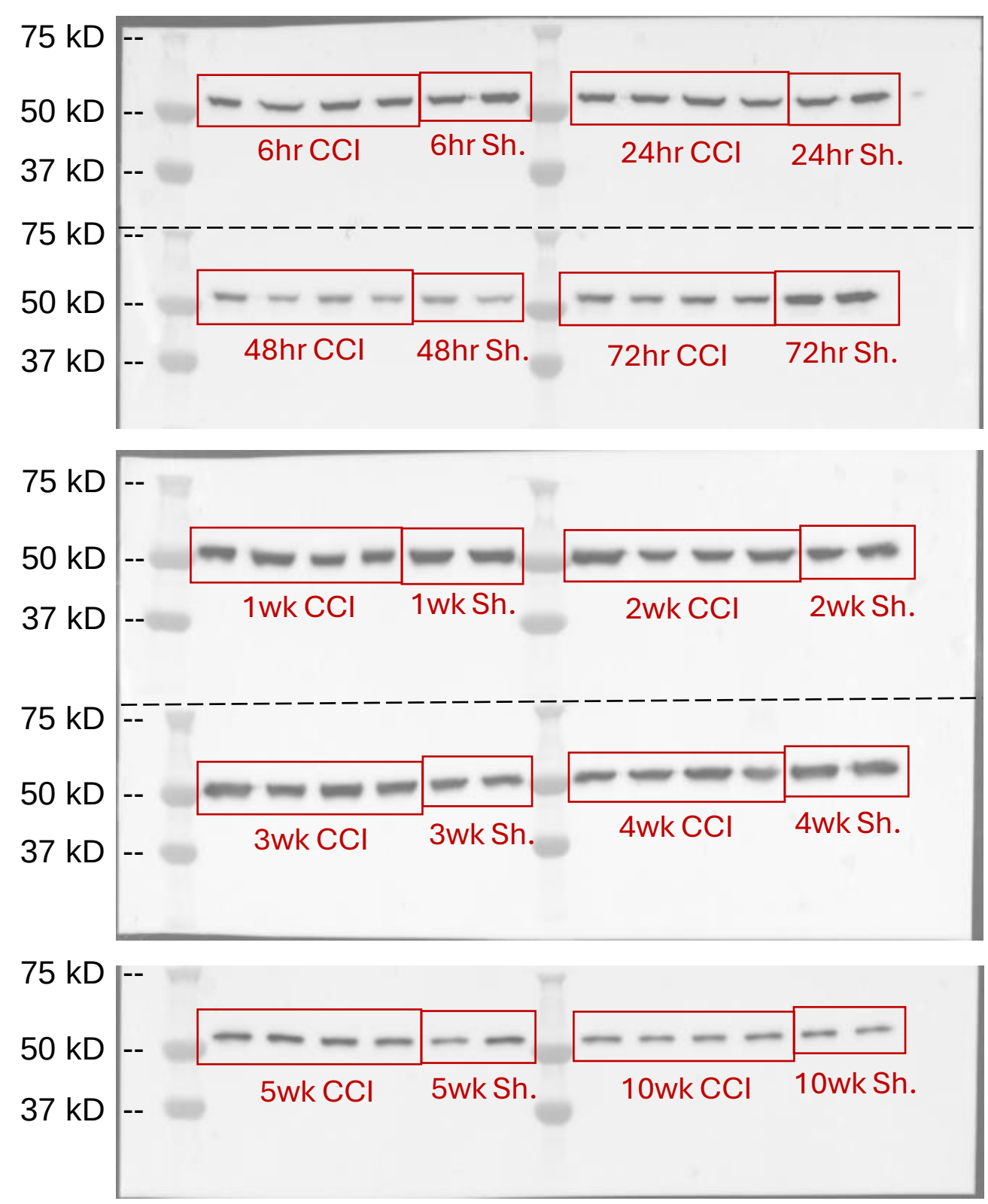

Supplement: Supplementary file 1 [file NRR-21-3620_Suppl1.pdf]
